# Heat-Damaged Fruits of ‘Čačanska Lepotica’ as a Raw Material for Plum Spirit Production: Possibilities for Improving Product Quality

**DOI:** 10.3390/foods15111855

**Published:** 2026-05-24

**Authors:** Branko Popović, Ninoslav Nikićević, Vele Tešević, Ivan Urošević, Olga Mitrović, Aleksandar Leposavić, Aleksandra Korićanac

**Affiliations:** 1 Fruit Research Institute, Čačak, Kralja Petra I 9, 32000 Čačak, Serbia; aleposavic@institut-cacak.org (A.L.); akoricanac@institut-cacak.org (A.K.); 2 Faculty of Agriculture, University of Belgrade, Nemanjina 6, 11080 Zemun, Serbia; ninoslavyug@yahoo.com; 3 Faculty of Chemistry, University of Belgrade, Studentski trg 12-16, 11000 Beograd, Serbia; vtesevic@chem.bg.ac.rs; 4 Faculty of Agriculture, Bijeljina University, Pavlovića put bb, 76300 Bijeljina, Bosnia and Herzegovina; ivanurosevic@tok.rs

**Keywords:** climate change, plum, processing, spirit drinks, volatile compounds, sensory characteristics

## Abstract

Because of the rising occurrence of extremely hot summers linked to climate change, there is a mass appearance of heat-damaged fruits of ‘Čačanska Lepotica’, which are unsuitable for fresh consumption and processing into products in which the fruits’ appearance is a critical quality attribute. To address this issue, the aim of the study was to examine the possibility of producing plum spirits from such fruits as one of the potential ways to utilize them. Heat-damaged fruits were processed in nine different ways, including a traditional one. All produced plum spirits met legal requirements. GC-MS analysis of 39 volatile compounds revealed that the plum spirits made from heat-damaged fruits had different aromatic profiles depending on the processing method applied. However, the plum spirit produced in the traditional way had various sensory defects; the most notable were a lack of freshness and a distinctly unpleasant ‘cooked plum’ aroma. Modifications to the production method led to an improvement in the sensory characteristics of the plum spirits. The obtained results indicate that modifications in small distilleries should include fruit crushing, stone removal and usage of baker’s yeast. In large distilleries, though, the best sensory characteristics for plum spirits made from heat-damaged fruits could be obtained using a production method that includes fruit pulping with stone removal and spontaneous alcoholic fermentation, with one additional step: lowering the pH of the mash before alcoholic fermentation to 3.0.

## 1. Introduction

Annual temperature variation from −25 °C to 35 °C enables the successful cultivation of European plum (*Prunus domestica* L.) in the conditions of Serbia and other European production regions [1]. However, all European plum-growing countries are affected by global climate changes. According to the cited authors [1], in the Čačak region, one of the most important plum production areas in Serbia, the average annual temperature during the 1965–2018 period increased by approximately 0.008 °C per year (from 9.69 °C in 1965 to 13.21 °C in 2018), and a similar pattern was observed for average temperatures in the vegetative cycle as well (from 14.45 °C in 1965 to 18.52 °C in 2018). As a result of climate change, a continuous increase in average temperatures during the 21st century is projected. Furthermore, extremely hot summers are among the phenomena expected to occur more frequently, which can have the direct impact of causing physiological changes in plants, leading to decreased yield and quality and also affecting plums [1,2]. Since the beginning of the 21st century, climate change has contributed to the emergence of previously unreported problems and phenomena in plum cultivation [3]. It has been observed that very high temperatures (above 35 °C) during fruit growth and ripening can result in plum fruit damage [1,3].

The appearance of massive damage in plum fruit in the Čačak region, as a result of the extremely high temperatures, was observed for the first time in 2007 on the fruits of the ‘Čačanska Lepotica’ variety. That year experienced a heat wave, with daily temperatures above 36 °C lasting between 17 and 24 July, with the absolute maximum temperature (43 °C) recorded on 24 July, and there was also the appearance of hot winds. This period of extreme heat coincided with the onset of the harvesting of plums intended for distant markets, whilst the harvest for the local market began 5–7 days later. However, just a few days after the end of the heat wave, a mass appearance of fruits with pronounced mesocarp browning was observed, making the plums completely unmarketable. This phenomenon had not been observed or described in the literature previously, nor did the producers have any experience with such fruit. A few years later, this phenomenon was also observed in other countries. Neumüller et al. [4] provided the first description of this phenomenon, assuming that browning of the fruit flesh was potentially associated with high temperatures during the later stages of fruit ripening. The authors observed that the browning of the fruit flesh occurred two days after the heat-wave period. In 2021, Neumüller et al. [3] described heat damage in the fruit flesh more comprehensively, considering its three different models of manifestation. According to the findings of the abovementioned authors, the ‘Čačanska Lepotica’ variety shows symptoms of heat damage Type-1 (‘flesh on one side of the fruit becomes softer and slightly vitreous; fruits have fermented taste; since being softer, affected fruits can be sorted out with considerable effort’). However, based on the symptoms previously observed in Serbia, the damage to ‘Čačanska Lepotica’ could instead be classified as Type-2 heat damage, explained by the previously mentioned authors in the following way: ‘there is no visible external damage; different extent of mesocarp browning; development of off-flavors; fruit become inedible’.

‘Čačanska Lepotica’ is grown in almost all plum-growing countries in Europe. In Serbia, which ranks among the four largest plum producers worldwide, this variety, along with ‘Čačanska Rodna’ and ‘Stanley’, is the dominant variety in orchards planted in the last 25 years [5]. ‘Čačanska Lepotica’ is a plum variety with combined properties; it is mainly used for fresh consumption but is also a very interesting raw material for the production of high-quality plum spirits with a fine, floral aroma [6,7]. However, to obtain top-quality fruit spirit, the fruit must be sound [8].

Traditional plum spirit production in European countries involves the spontaneous alcoholic fermentation of whole or slightly crushed fruits with stones, followed by distillation of the fermented mash. In the case of plum varieties with larger fruits, plums should be crushed without removing the stones, while in the case of varieties with smaller fruits, stone removal is recommended [9]. ‘Čačanska Lepotica’ is characterized by medium-to-large-sized fruits [10]. To further improve the quality of plum spirits, the use of commercial enzyme preparations, lowering the pH of the mash by acids, and distillation of the fermented mash immediately after fermentation are also recommended [9]. Our previous works indicate that the quality of plum spirits, especially their sensory characteristics, can be significantly changed by modification of the traditional production method, although the extent of quality changes depends on the distillery equipment and cultivar. High-quality plum spirits can be produced from certain varieties using traditional methods, whereas other varieties require modifications to the production method [11]. Stone removal during processing of ‘Čačanska Lepotica’ results in better sensory quality of plum spirits since the aromatic substances originating from the stones are not compatible with the fine, delicate fruit–floral aromas of the components originating from the fruit or formed during fermentation [6]. Previous studies showed that the use of the traditional method in plum spirit production from ‘Čačanska Lepotica’ resulted in plum spirits with lower sensory quality than those obtained in the same way from other plum varieties [12,13,14].

To address the issue of heat-damaged plums, the goal of this work was to examine the potential to produce high-quality plum spirits from heat-damaged fruits in the traditional way and, furthermore, to determine whether the quality of the distillate can be improved by modifying the technological production process in the predistillation phase.

## 2. Materials and Methods

### 2.1. Raw Material

#### 2.1.1. Plum Fruit Harvest

Fully ripe plums, at the standard ripening stage for plum spirit production, were harvested from two localities, whose altitudes and coordinates are shown in Table 1. All usual agro-technical measures, except irrigation, were applied in the orchards at both localities.

**Figure 1:**
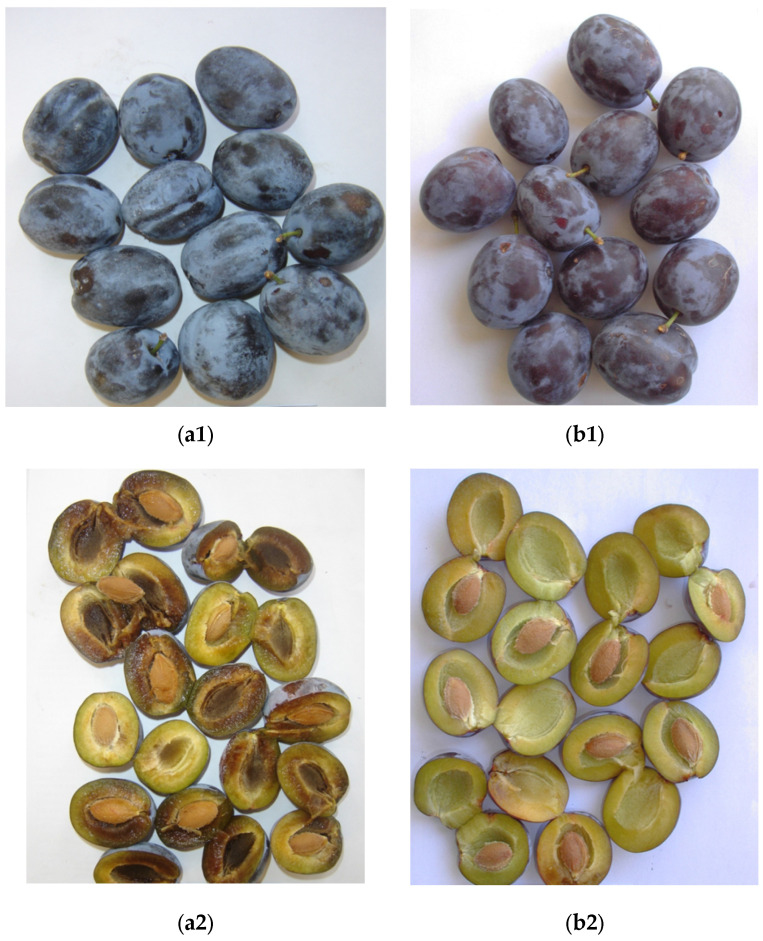

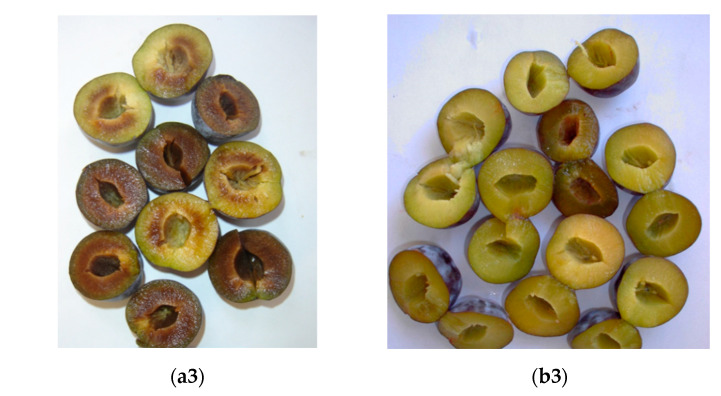
‘Čačanska Lepotica’ fruits. (**a1**–**a3**) Heat-damaged fruits from Preljina locality; (**b1**–**b3**) Sound fruits from Gledjica locality.

Heat-damaged fruits were collected on 9 July 2007, during the morning hours at the locality of Preljina (P), 10 km away from Čačak (20 randomly selected trees were completely harvested—about 600 kg of fruit), and on 17 July 2007, sound, undamaged fruits were collected from another locality, Gledjica (G), 80 km away from Čačak (6 randomly selected trees were completely picked—about 180 kg of fruit). Harvests were carried out in 2007, when heat-damaged fruits were observed for the first time in the Čačak region. Almost all fruits harvested at the locality of Preljina (P), in the Čačak region plum-growing zone, had visible heat-damage symptoms in the fruit flesh, although their external appearance did not differ from sound fruits taken from the second locality (G) (Figure 1).

#### 2.1.2. Meteorological Data

The locality of Preljina, from which the heat-damaged fruits were taken, is located in the Čačak region. The following climatic variables, obtained from the Republic Hydrometeorological Service of Serbia (Figures S1 and S2, Supplementary Materials), were observed in the Čačak region over a 25-year period (2000–2024): average monthly air temperatures (°C), maximum daily air temperatures (°C), number of days with maximum daily air temperatures ≥ 30 °C, and the monthly sunshine duration (h) in July and August (months in which the fruits of ‘Čačanska Lepotica’ ripen). The same data source was used for maximum daily temperatures during July and August 2007 (Figure S3, Supplementary Materials), during which the appearance of heat-damaged fruit in ‘Čačanska Lepotica’ was observed for the first time.

#### 2.1.3. Basic Characteristics of Fruit for Plum Spirit Production

The basic characteristics of heat-damaged fruit (from the Preljina locality) and sound fruit (from the Gledjica locality) are shown in Table 1. For the analysis, 50 randomly selected heat-damaged or sound fruits were selected. Fruit and stone weights were measured using a Mettler technical scale balance, and the stone ratio in the fruit was determined afterwards. The following methods [15,16] were used for the chemical analysis of the fruit: soluble solids content was determined refractometrically (3828 manual refractometer, Carl Zeis, Jena, Germany), total sugars content by the Luff–Schoorl method, total acids content by the titrimetric method (0.1 M NaOH and phenolphtalein; the total acid content was expressed as malic acid (%) on a fresh weight basis), contents of individual fractions of pectin substances (pectinic acid—water soluble fraction, pectic acid—ammonium oxalate soluble fraction, protopectin—alkali-soluble fraction) by the carbazole method, and pH value potentiometrically (pH meter Mettler Toledo EL 20-Basic, Schwerzenbach, Switzerland). Soluble solids/total acids, total sugars/total acids, pectinic acid/protopectin and total sugars/total pectins ratios were calculated. The values of the examined parameters were within the range for the fully ripe and slightly overripe fruits of ‘Čačanska Lepotica’, which are the usual ripening stages of plums used for plum spirit production [17].

### 2.2. Plum Spirit Production at the Pilot Scale

#### 2.2.1. Predistillation Steps (Fruit Processing and Fermentation)

The sound, undamaged fruits (locality Gledjica) were processed using two methods, and the heat-damaged fruits (locality Preljina) were processed using nine methods. The modifications to the production method, which included various steps in the predistillation phase, are shown in Table 2.

For the variants in which the plums were fermented with stones (G1, P1, P3, and P5), crushed plum mashes with stones were obtained using a toothed roller crusher [11]; the fruits were broken, but without stone damage. If crushed plum mashes without stones (G2, P2, P4, and P6) were prepared for fermentation, the stones were removed manually; the obtained mashes had similar physical characteristics [11] to the mashes with stones (the mashes contained large pieces of plums, i.e., whole plum halves). For the variants (P7, P8, and P9) that included using a pilot pulping machine (capacity, 350 kg/h; perforations of the sieve, 8 mm), pulped mashes without stones (with a liquid consistency) were obtained along with simultaneous separation of the stones [11].

With the exception of variant P9, for which the plum mash pH was adjusted to pH 3 by the addition of sulfuric acid (1:10), in all other variants (G1–G2 and P1–P8), alcoholic fermentation was carried out at the natural pH of the plums.

Spontaneous alcoholic fermentation (SAF) was carried out for variants G1–G2, P1–P4, P7 and P9. Fresh baker’s yeast *Saccharomyces cerevisiae* (Vrenje, Serbia) was used (100 g/100 kg mash) for alcoholic fermentation for variants P5, P6 and P8. Before adding, the baker’s yeast was activated for 10 min in water at a temperature of 35 °C.

For all variants, alcoholic fermentations were carried out in triplicate (in each of 3 vessels made of polyethylene, 20 kg of the plum mash was placed). Alcoholic fermentation was carried out traditionally in open vessels, in which the surface of the mash was in constant contact with air. The temperatures of the mashes during alcoholic fermentations were 22 ± 2 °C. During alcoholic fermentation, decreases in the content of soluble solids in the mash were monitored daily using a manual refractometer (model 3828, Carl Zeis, Jena, Germany) (Figure S4, Supplementary Materials).

With the exception of variants P3 and P4, for which the fermented mashes were distilled after 15 days of storage, the fermented mashes obtained from the other variants were distilled immediately after the completion of alcoholic fermentation.

#### 2.2.2. Distillation

A pilot-scale traditionally constructed alembic made of copper, with a volume of 25 L, was used for the distillation of the fermented mashes as well as for the redistillation of the first distillate. The boiler was heated with a gas burner by an open flame. The first distillates with an ethanol content of 28.0 ± 0.3% *v*/*v* were obtained by the distillation of the mashes. The yields of the first distillates (obtained in three replicates for each variant separately) were summed, divided by 3 and then multiplied by 5 to express the average distillate yield (in L 28% *v*/*v* distillate/100 kg of mash). The first distillates, from three replicates for each variant, were mixed and then redistilled in the same alembic. During redistillation, the first fraction (head) was separated in the amount of 1% of the first distillate placed in the boiler of the alembic, and the middle fraction (heart) was separated from the last fraction (tail) when the ethanol content in the middle fraction was 60.0 ± 0.3% *v*/*v*. Only the middle fraction (heart) was used for further analyses.

### 2.3. Plum Spirit Analysis

#### 2.3.1. GC-FID Analysis of Major Volatile Compounds

The major volatile components were analyzed based on the European Community Reference Methods for the Analysis of Spirits using gas chromatography (GC) with a flame-ionization detector (FID) [18]. The main components, including methanol, acetaldehyde, 1-propanol, ethyl acetate, 2-methyl-1-propanol, 1-butanol, amyl alcohols and 1-hexanol, were identified by comparing their retention times with those of authentic compounds. The GC analysis was performed with an HP 5890 II gas chromatograph equipped with a flame ionization detector (FID) and a split/splitless injector (Hewlett-Packard Company, Wilmington, DE, USA). A capillary column (30 m × 0.25 mm i.d., 0.25 µm film thickness) coated with HP-5 MS (5% biphenyl and 95% dimethylpolysiloxane) was used. The column oven temperature was programmed from 50 °C to 285 °C at a rate of 4.3 °C/min, and the injection port and detector temperatures were maintained at 250 °C. Hydrogen was used as the carrier gas at a flow rate of l.6 mL/min, and the split ratio was 60:1. The sample injection volume was 1 µL.

#### 2.3.2. Spectrophotometric Analysis of HCN

HCN (hydrocyanic acid) content in the plum spirits was determined by the spectrophotometric method described by Spaho et al. [19], using a Beckman spectrophotometer model 24 (Brea, CA, USA). HCN from the plum spirit samples was converted by 1% chloramine T into cyanogen chloride. Phosphate buffer (pH 7.6) was used to adjust the pH value. Cyanogen chloride produced a violet-blue coloration after the addition of a reactive solution consisting of 1,3-dimethylbarbituric acid and pyridine. The absorbance of the formed colored complex was measured at a wavelength of 584 nm and then compared to the colors of the standard solutions. The contents of HCN in the plum spirits were expressed as mg/L a.a. (milligrams per liter of absolute alcohol).

#### 2.3.3. Volumetric Analysis of Total Acids and Total Esters

Titration with 0.1 M NaOH was applied to measure total acids with a phenolphthalein indicator. Total acids were expressed as mg/L a.a. of acetic acid (milligrams per liter of absolute alcohol) [20].

Total esters were quantified through saponification of esters in the distillate with standard 0.1 M NaOH solution, followed by titration with standard 0.05 M H_2_SO_4_ solution, and expressed as ethyl acetate equivalents mg/L a.a. [20].

#### 2.3.4. GC-MS Analysis of Volatile Compounds

All samples were prepared by liquid–liquid extraction, with three replicates for each sample. Fifty milliliters of distillate was mixed with 100 mL of ultrapure water, and 20 mL of internal standard (1 mg/mL, methyl 10-undecenoate) was added; then, the mixture was extracted with 20 mL of dichloromethane and NaCl (10 g). The samples were then mixed for 1 h on magnetic stirrers in closed ground Erlenmeyer flasks for 30 min. Layers were separated in a separator funnel, and the organic layer was dried over anhydrous sodium sulfate, filtered, evaporated to a volume of 2 mL, and then transferred to a 2.5 mL vial. Evaporation was performed on a vacuum evaporator, with the water bath filled with cold water.

The purity of the standard was confirmed by GC/FID and by infrared spectroscopy (it was shown to contain no water, as it was not visible by GC/FID).

GC/MS analysis was performed using an Agilent 6890 gas chromatograph coupled with an Agilent 5973 Network mass selective detector (MSD) (Santa Clara, CA, USA) operated in positive ion electron impact (EI) mode. The separation was achieved on an Agilent 19091N-113 (Santa Clara, CA, USA) fused silica capillary column, 30 m × 320 μm × 0.25 μm, with a polar liquid phase HP-INNOWax (polyethylene glycol). The GC oven temperature was programmed from 40 to 230 °C at a rate of 3 °C/min. Helium was used as the carrier gas, the inlet pressure was 25 kPa, and the velocity was 1 mL/min at 210 °C. The injector temperature was 250 °C, and the injection mode was splitless. FID and MSD were used simultaneously as detectors. The MS scan conditions were a source temperature of 200 °C and an interface temperature of 250 °C; the energy of the electron beam was 70 eV, and the mass scan range was 40–350 amu (atomic mass units). The identification of the components was based on retention indices and comparison with reference spectra (Wiley and NIST databases). The percentages of the identified compounds were computed from the GC peak areas according to the following formula:

C = 20 × Vs × Cs × P/Ps

C—The concentration of the substance in the sample expressed in mg/L;
Cs—Standard concentration expressed in mg/mL;
Vs—Volume of added standard expressed in mL;
P—Sample peak area;
Ps—Peak area of the standard;
20—Factor for converting the concentration to 1 L, since the volume of the sample is 50 mL.

The contents of individual components are expressed in mg/L (milligrams per liter). Odor activity values (OAVs) for individual volatile compounds were calculated as quotients of their concentration in plum spirits and their odor thresholds (OTs) given in the literature [21,22,23,24,25,26,27,28,29,30].

#### 2.3.5. Sensory Analysis

A five-member, highly experienced panel performed sensory analysis of the plum spirits by the Buxbaum method [11]. Previously, the heart fractions obtained by the second distillation were diluted with deionized water to make them drinkable (ethanol contents were reduced from 60.0 ± 0.3 to 45 ± 0.3% *v*/*v*). Color (maximum 2 points), clarity (maximum 1 point), odor (maximum 7 points), and taste (maximum 10 points) were evaluated, and each plum spirit could obtain a maximum of 20 points in total. Plum spirits with ≤14.01 points were not awarded a medal; those with 14.01–16.00 points received a bronze medal; those with 16.01–18.00 received a silver medal; and those with 18.01–20.00 received a gold medal.

### 2.4. Statistical Analyses

Statistical analyses were performed using Statistica 7 software (StatSoft Inc., Tulsa, OK, USA). One-way ANOVA and Tukey’s test (*p* < 0.05) were used for the determination of existing significant differences: (i) among yields of plum spirits produced by different methods from the sound and heat-damaged plums picked in two localities; (ii) among concentrations of volatile compounds analyzed by GC-MS in the plum spirits; and (iii) among the sensory ratings of these plum spirits. Principal component analysis (PCA) based on the content of 39 volatile compounds (12 higher alcohols, 12 esters, 6 aldehydes, 4 acetals, and 5 volatile acids) was carried out by the same software.

## 3. Results and Discussion

### 3.1. Key Climate Variables and Occurrence of Heat-Damaged Fruits

Climate variables during July and August, when ‘Čačanska Lepotica’ ripens, were analyzed for the first quarter of the 21st century (Figures S1 and S2; Supplementary Materials). In 2007, their values were significantly higher than the average for the Čačak area (Preljina locality). Although not systematically studied, we observed a connection between the appearance of heat-damaged plum fruit and the selected climate variables in July (Figure S1). More or less pronounced symptoms of heat damage among the fruit of ‘Čačanska Lepotica’ were observed in the Preljina locality when the values of all selected climate variables were extremely high in July for the given region: the average monthly air temperature was ≥24 °C, the maximum daily air temperature was ≥39 °C, the number of days with maximum daily air temperatures ≥ 30 °C were ≥20, and the monthly sunshine durations were ≥330 h. No relationship was observed between heat-damaged fruits and the selected climate variables in August (Figure S2), which is expected since ripening of ‘Čačanska Lepotica’ mainly occurred in the second half of July. Such extreme climatic factors were also recorded in 2012, 2022 and 2024 (Figure S1). Although the experiment was not conducted in the aforementioned years, significant occurrences of heat-damaged fruits of ‘Čačanska Lepotica’ were reported by plum growers and plum spirit producers, based on personal observations. Additionally, the plum spirits produced in these years using traditional methods were often characterized by a ‘cooked plum’ aroma.

The orchard in Preljina (P) is located at 390 m (Table 1), within the ideal altitude range (200–700 m) for plum growing in Serbia [10]. Heat-damaged fruits of ‘Čačanska Lepotica’ were harvested from this orchard. Plums can be grown at altitudes up to about 1000 m. According to these authors [10], the air temperature drops by 0.5 °C for every 100 m increase in altitude, reducing the possibility of heat damage. Therefore, sound, undamaged plums were harvested from an orchard located at the Gleđica (G) locality at an altitude of 790 m. Plums at the same ripening stage were harvested 8 days earlier at the Preljina locality than at the Gledjica locality, which is expected since certain phenophases occur 1–3 days later with an increase in altitude by 100 m. The contents of soluble solids and total sugars were not significantly reduced in heat-damaged fruits, but the visual symptoms (flesh browning) as well as the pronounced ‘cooked’ taste of the fruits from the Preljina locality indicated heat damage Type-2, as described by Neumüller et al. [3].

### 3.2. Plum Distillate Yields

Obtaining a high distillate yield from a certain amount of fruit is one of the most important requirements when considering the suitability of a raw material for processing into a spirit drink [8]. Contrary to the findings of Neumüller et al. [3], we found no significant difference in total sugar content between sound fruits (12.45%) and heat-damaged fruits (11.70%) of ‘Čačanska Lepotica’.

**Figure 2:**
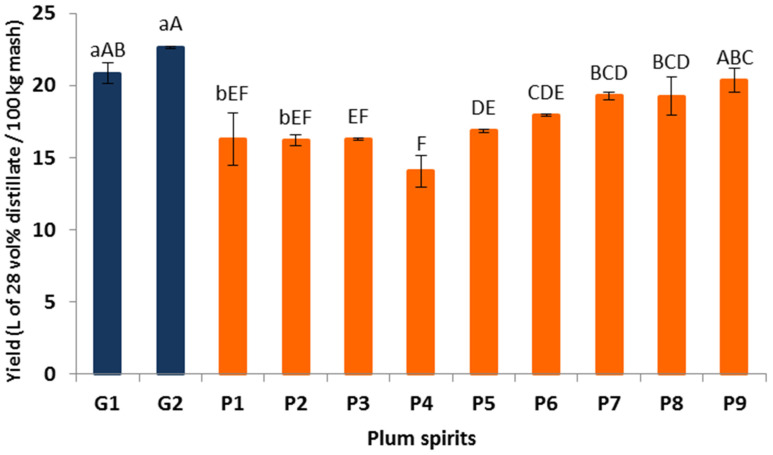
Yield of distillates obtained from sound fruits (G1–G2) and heat-damaged fruits (P1–P9). Different lowercase letters indicate statistically significant differences among the four samples (G1, G2, P1, and P2) produced from sound and heat-damaged fruits of ‘Čačanska Lepotica’ (Tukey’s test, *p* < 0.05). Different uppercase letters in the same row indicate statistically significant differences among all plum spirit samples (G1–G2 and P1–P9) (Tukey’s test, *p* < 0.05). Ratings of all plum spirits are shown as mean ± standard deviation.

For the plums taken from the two localities that were processed in the same way (comparison of plum spirits G1 and G2 with samples P1 and P2), significantly lower yields of distillates were obtained from heat-damaged plums than from sound, undamaged ones—by 22.03% when comparing P1 and G1 and 28.51% when comparing P2 and G2 (Figure 2). The significant reduction in distillate yield when processing heat-damaged plums could not be entirely attributed to the slightly lower sugar content, which was reduced by only 6.03% in the unhealthy fruits. This decrease was likely influenced by differences in the physical characteristics of the plums and differences in the indigenous microbial flora of the fruits from the two localities. However, by modifying the processing methods of heat-damaged plums, the yield of plum spirit was significantly changed (Figure 2). In the case of the fermented mash stored for 15 days before distillation, the distillate yield was reduced by 13.54% (comparison of distillate yields obtained by methods P4 and P2). The modified method of processing heat-damaged plums, which included plum pulping, lowering the plum mash pH to 3.0, and spontaneous fermentation followed by distillation immediately after the completion of fermentation (P9), resulted in a significant increase in the distillate yield compared to that obtained from crushed plums (P2) by 25.49% and significantly approached the yields of distillates obtained from sound plums from the Gledjica locality (G1 and G2). In general, modifications to the heat-damaged plum processing methods, which included the distillation of the mash immediately after fermentation, adding baker’s yeast in the fermentation of crushed plums, and pulping of the fruits (regardless of the fermentation type), led to an increase in the yield of plum distillate (methods P5–P9 compared with methods P1–P4). Modifications to production methods P5–P9 affected the distillate yield in the following ways: they created favorable conditions for the activity of ellipsoidal yeasts, which are important for successful alcoholic fermentation, while reducing the growth and activity of undesirable acetic acid bacteria and lactic acid bacteria that regularly occur during fermentation in the open vessels commonly used in Serbia. These bacteria can use alcohol formed by yeasts or compete with yeasts for sugar as a substrate for alcoholic fermentation, leading to significant reductions in distillate yields [11,31,32].

### 3.3. Compliance of Plum Spirit Quality with Legal Requirements

All analyzed plum spirits from both sound and heat-damaged fruits met EU legal requirements [33] in terms of potentially hazardous methanol and HCN, as well as the volatile substances used for the determination of spirit authenticity (Figure S5, Supplementary Materials).

Besides the GC-FID method, titrimetric methods are also used to demonstrate the compliance of these products’ compositions with past regulations [34,35,36], as well as to preliminarily evaluate the sensory acceptability of spirit drinks. Figure S6 (Supplementary Materials) shows that all produced plum spirits from ‘Čačanska Lepotica’, regardless of the fruit health status and the processing method applied, were aligned with the regulation requirements in terms of total acid and total ester contents. The highest contents of total acids and total esters were found in the samples produced by distillation of the fermented mashes that were stored 15 days after the completion of alcoholic fermentation—samples P3 (1920 mg/L a.a. of total acids and 3867 mg/L a.a. of total esters) and P4 (1805 mg/L a.a. of total acids and 3927 mg/L a.a. of total esters). Plum spirits with more than 1600 mg/L a.a. of total acids [37], particularly due to the predominant presence of acetic acid with its distinctly sour taste, may be perceived as excessively sour, which is generally unacceptable to modern consumers. Furthermore, when the total esters content exceeds 3000 mg/L a.a., the plum spirit exhibits a pronounced ester aroma characterized by solvent- or glue-like notes. GC-FID analysis showed (Figure S7, Supplementary Materials) that the content of ethyl acetate in plum spirit samples P3 (2883 mg/L a.a.) and P4 (2992 mg/L a.a.) was above 2180 mg/L a.a., the threshold concentration set by Scholten and Kacprowski [38], above which a more or less pronounced unpleasant solvent-like odor appears.

In addition to ethyl acetate, other components analyzed by GC-FID (Figure S7, Supplementary Materials) may positively influence the sensory characteristics of spirit drinks by contributing body and fruity notes when present at lower concentrations. However, they show a negative effect if the following limit values are exceeded [38,39,40,41]: 125 mg/L a.a. (acetaldehyde—sharp, pungent, green apple-like aroma), 1620 mg/L a.a. (1-propanol—solvent, fusel note), 2900 mg/L a.a. (2/3-methyl-1-butanol—heavy, fusel note) and 100 mg/L a.a. (1-hexanol—green, grassy, herbaceous). The concentrations of some of the abovementioned compounds were above the limit values only in a few plum spirits produced from heat-damaged fruits: acetaldehyde (in samples P2 and P8), 1-propanol (P5 and P6), and 1-hexanol (P1, P3, and P4).

In all plum spirits obtained from the spontaneously fermented mashes, the predominant higher alcohols were isoamyl alcohols (the sum of 2- and 3-methyl-1-butanol) (Figure S7, Supplementary Materials). 1-Propanol was dominant only in the plum spirits obtained from mashes fermented by baker’s yeast (P5, P6 and P8). The IA/1P ratios (isoamyl alcohol—IA and 1-propanol—1P) in the plum spirits obtained by spontaneous fermentation of the mashes were ≥1.00 and <1.00 in the samples where baker’s yeast was used for alcoholic fermentation (Table 3), which is in accordance with the results of Pielech-Przybylska et al. [42]. Regardless of the plum health status and fruit processing method, the IA/IB ratios were >1, and the IB/1P ratios were <1 in all plum spirits, which is also in accordance with the cited study [42].

### 3.4. GC-MS Analyses of ‘Čačanska Lepotica’ Plum Spirits Produced from Sound and Heat-Damaged Fruits

GC-MS analysis is commonly used to determine differences in volatile compounds in plum spirits based on geographical origin, plum variety, and processing method [43,44,45]. The number of quantified compounds can vary due to differences in the GC-MS methods employed. A total of 39 compounds (12 alcohols, 12 esters, six aldehydes, four acetals and five acids) were quantitatively analyzed in plum spirits from ‘Čačanska Lepotica’ (from both heat-damaged and sound fruits) (Table 4). Vyvurska et al. [43] reported that among 124 compounds identified in plum spirits from 25 varieties produced in the same way, the one derived from ‘Čačanska Lepotica’ contained only 74 compounds. According to Satora et al. [13], among the 44 quantified volatile compounds, plum spirit from ‘Čačanska Lepotica’ contained significantly more ethyl cinnamate and less benzyl acetate, ethyl tetradecanoate, and α-ocimene than plum spirits from other cultivars.

Among the components of all plum spirits analyzed by the GC-MS method, the following originated from the raw material: 1-hexanol, *Z*-3-hexenol, 1-heptanol, 1-octanol, 1-nonanol, benzyl alcohol, ethyl benzoate, ethyl cinnamate, hexanal, heptanal, nonanal, furfural, benzaldehyde, 1,1-diethoxy hexane and 1,1-diethoxy nonane [17,30]. Other compounds detected in plum spirit samples (six higher alcohols, 10 esters, acetaldehyde, and five acids) were produced by yeasts and other microorganisms mainly during alcoholic fermentation [30,31,32,41,46].

Modifying certain predistillation steps during plum spirit production significantly affects its composition [11,44]. The GC-MS analysis results indicated that the same also applies to the processing of heat-damaged fruits of ‘Čačanska Lepotica’.

#### 3.4.1. Effect of the Health Status of Plums on the Composition of Plum Spirits Made from Crushed Mash

One-way ANOVA (Table 4) was applied only to the plum spirits (two from sound fruits and two from heat-damaged fruits of ‘Čačanska Lepotica’) obtained from spontaneously fermented crushed plums with or without stones (samples G1, G2, P1 and P2), since the physical characteristics of the plum mashes obtained by procedures G1 and G2, or P1 and P2, can be considered similar. It was found that the plum spirits from heat-damaged fruits (P1 and P2) contained significantly lower concentrations of 1-propanol, 2-methyl-1-propanol, 2/3-methyl-1-butanol, ethyl octanoate, ethyl decanoate, propyl acetate, ethyl cinnamate, and furfural than those obtained from sound fruits (G1 and G2). At the same time, the plum spirits from heat-damaged fruits (P1 and P2) contained significantly higher concentrations of 1-hexanol, *Z*-3-hexenol, 1-nonanol, hexanal, heptanal, 1,1-diethoxyhexane, and 1,1-diethoxynonane. A similar result was obtained by application of one-way ANOVA to all eleven plum spirit samples. The results also indicated that the plum spirits from heat-damaged fruits (P1 and P2) contained more ethyl lactate than those from sound fruits (G1 and G2). The observed variations most likely originated from the differences in the compositions of sound plums and heat-damaged plums. Variable contents of higher alcohols (1-propanol, 2-methyl-1-propanol, 2/3-methyl-1-butanol) and esters (ethyl octanoate, ethyl decanoate, propyl acetate), primarily formed by the metabolic activity of yeasts, can likely be attributed to the different amino acid compositions [47] of fruits with different health statuses. However, the influence of location itself on the differences in chemical composition of plums should not be excluded [48]. Moreover, differences in the indigenous microbial flora of plum fruits from different localities may contribute to the observed variations [49]. On the other hand, higher contents of ethyl cinnamate in plum spirits from sound fruits may indicate a better preservation of this ester in undamaged fruits, which is important for the aroma of plums and plum spirits [13,50]. The higher contents of 1-hexanol, *Z*-3-hexenol, 1-nonanol, hexanal, heptanal, 1,1-diethoxy hexane, and 1,1-diethoxy nonane, which primarily originate from the raw material, in plum spirits made from heat-damaged fruits suggest that the lipoxygenase pathway was induced in the damaged tissue.

#### 3.4.2. Effect of Stone Presence in the Mash as a Factor of the Quality of Plum Spirits Made from Crushed Sound or Heat-Damaged Fruits

The simplest modification to the traditional plum spirit production method is stone removal, which is carried out in order to improve the “health” value and sensory characteristics of plum spirits from certain plum varieties [6,51,52]. The stoneless plum mashes had similar physical characteristics as the plum mashes with stones (comparison of G1 and G2 when processing sound plums, i.e., comparison of pairs P1 and P2, P3 and P4, as well as P5 and P6 in heat-damaged plums). Among the plum spirit samples in all the pairs considered, the same pattern was observed only for compounds whose occurrence in the product is directly associated with the presence of stones in the mash (benzaldehyde and benzyl alcohol). In other words, the plum spirit produced from the stoneless mash contained a lower amount of these ingredients than the plum spirit produced from the mash with stones (Table 4). Our earlier work showed that regardless of variety, a plum spirit with a significantly lower benzaldehyde content was obtained from a crushed plum mash without stones than from a crushed plum mash with stones [6]. In addition, similar patterns were observed for ethyl propanoate and ethyl lactate, which indicates a cleaner alcoholic fermentation and lower bacterial activity in stoneless mashes. Consequently, lower amounts of these esters were found in the corresponding plum spirits produced from stoneless mashes.

#### 3.4.3. Effect of the Storage Time of Fermented Mash from Crushed Heat-Damaged Plums

Storage of fermented mash in open vessels for 15 days, a characteristic of traditional plum spirit production in Serbia, can have an opposite effect on the quality of plum spirits depending on the variety. It can enhance the sensory characteristics of plum spirits made from certain plum varieties due to the increased complexity of their compositions. However, in other varieties, it may have a negative effect due to reduced fineness of the aroma [51]. The 15-day storage of the fermented mash led to a significant increase in the content of numerous components (both when comparing P3 with P1 and when comparing P4 with P2): five alcohols (2-methyl-1-propanol, 1-butanol, 2/3-methyl-1-butanol, 1-pentanol, and 1-nonanol), eight esters (ethyl acetate, ethyl propanoate, ethyl hexanoate, ethyl octanoate, ethyl decanoate, ethyl lactate, methyl acetate, and propyl acetate), and acetic acid (Table 4). A significant increase in the contents of benzyl alcohol, ethyl benzoate and benzaldehyde in the plum spirit was observed only when processing the plums with stones (comparison P3 with P1). Storage of fermented plum mash in open vessels provides suitable conditions for the development of acetic acid bacteria and lactic acid bacteria, leading to increased contents of ethyl acetate, acetic acid and ethyl lactate [11]. These effects were also indicated by volumetric analysis of the contents of total esters and total acids in the plum spirit samples (Figure S6). The increased content of propyl acetate and methyl acetate corresponded to an increased amount of the alcohols and acetic acid (which are the basic substrates for the formation of these esters) in the plum spirits from the fermented mashes stored for 15 days. Extended storage of the fermented plum mash with stones (comparison P3 with P1) led to higher concentrations of compounds formed by the enzymatic degradation of cyanogenic glycoside amygdalin [53], such as benzaldehyde and its derivatives (benzyl alcohol and ethyl benzoate), in the plum spirits.

#### 3.4.4. Effect of Fresh Baker’s Yeast Addition in Crushed Plum Mash from Heat-Damaged Fruits

Use of selected yeasts in the production of plum spirits enables better control of alcoholic fermentation and modulation of the product chemical composition; the addition of yeast does not necessarily result in a product with improved sensory properties, though [11,42,44]. Although there was no visible damage on the outside, it is possible that the epiphytic indigenous microbial flora on heat-damaged fruits of ‘Čačanska Lepotica’ was altered. Therefore, yeast was added to control the fermentation process. The fresh baker’s yeast used in this study is inexpensive and widely available. Intended for bakeries, it could also serve as a viable alternative to active dry yeast strains for distilleries, which are less accessible and typically available only in specialized stores. Recent scientific studies on the production of spirit drinks have been carried out with yeast strains that were not originally intended for their production [54]. Therefore, we compared the composition of plum spirits (Table 4) obtained by using baker’s yeast (P5 and P6) with those obtained by spontaneous fermentation (samples P1 and P2). In both cases, the fermented mashes were distilled immediately after the end of fermentation. The plum spirits produced with baker’s yeast (P5 and P6) had significantly higher levels of components that are mainly considered to be products of yeast metabolism (1-propanol, 2-methyl-1-propanol, 1-butanol, 2/3-methyl-1-butanol, ethyl hexanoate, ethyl octanoate, ethyl decanoate, propyl acetate, isoamyl acetate, phenylethyl acetate, hexanoic acid, octanoic acid, decanoic acid and dodecanoic acid). The effect of the yeast on the concentrations of 1-heptanol and 1-octanol was similar. Additionally, the content of benzyl alcohol was higher in the plum spirit obtained using baker’s yeast (only for plum processing methods with stones—comparing P5 with P1). On the other hand, the use of baker’s yeast in the P5 and P6 plum spirits caused significantly lower contents of ethyl acetate, ethyl lactate, and acetic acid, products of the activity of non-*Saccharomyces* yeasts and acetic acid and lactic acid bacteria present in the complex microbial flora during spontaneous alcoholic fermentation. Additionally, significantly lower levels of furfural were found in samples P5 and P6 than in P1 and P2. Furthermore, samples P5 and P6 contained lower contents of methyl acetate, hexanal, heptanal, 1,1-ethoxy methoxy ethane, and 1,1-diethoxy ethane due to the rapid completion of fermentations in samples with added yeast. In other words, the fermentation duration was too short to allow the formation of these compounds from precursors present in plums through specific enzymatic reactions. These results are consistent with previous studies [49,55] that compared the composition of plum spirits obtained using selected strains of *Saccharomyces cerevisiae* and plum spirits obtained by spontaneous fermentation.

#### 3.4.5. Effect of Heat-Damaged Plum Pulping (With/Without Baker’s Yeast Addition and pH Lowering)

The plum spirit obtained from heat-damaged fruits by the spontaneous fermentation of pulped plum mash distilled immediately after fermentation (P7) contained significantly higher amounts of 1-propanol, 2-methyl-1-propanol, 1-butanol, 2/3-methyl-1-butanol, 1-pentanol, 1-hexanol, *Z*-3-hexenol, 1-heptanol, ethyl hexanoate, ethyl octanoate, ethyl decanoate, isoamyl acetate, phenylethyl acetate, 1,1-diethoxy hexane, hexanoic acid, octanoic acid, decanoic acid and dodecanoic acid, and significantly lower amounts of 1-octanol, 1-nonanol, benzyl alcohol, ethyl acetate, ethyl lactate, ethyl benzoate, methyl acetate, propyl acetate, acetaldehyde, hexanal, furfural, heptanal, 1,1-ethoxy methoxy ethane, 1,1-diethoxy ethane, and acetic acid, compared to the plum spirits obtained from the same raw material but produced by spontaneous fermentation of the stoneless crushed plum mash (P2). When the pulping of heat-damaged fruits was followed by lowering the pulped plum mash pH (from the native value to pH 3.0), the changes in the content of the plum spirit components occurred (comparison of the samples P9 with P2) in almost an identical way as those in the comparison of samples P7 and P2. In both cases (comparison of P7 and P2, i.e., P9 and P2), the changes in the physical characteristics of the mashes probably contributed to the different growth dynamics of members of indigenous microbial flora during alcoholic fermentation, thus obtaining plum spirits with a higher content of compounds formed by ellipsoidal yeast [11,55]. Also, pulping contributed to the faster establishment of contact between the substrate (polyunsaturated fatty acids) and the lipoxygenase pathway enzymes, thus increasing the content of the products (e.g., C6 alcohols) of this pathway in plum spirits.

However, in contrast to the above two cases, pulping of fruits followed by the addition of baker’s yeast did not cause such significant changes in the composition of the plum spirit compared to the plum spirit made from the crushed stoneless mash that was also fermented with baker’s yeast (comparison P8 with P6). Since the same yeast was used in both the P8 and P6 samples, no significant differences were observed in the content of those components that are primarily produced by the metabolic activity of yeasts (e.g., higher alcohols, esters of higher fatty acids, volatile fatty acids), as well as those that are indicators of the development of undesirable bacteria (e.g., ethyl acetate, ethyl lactate). The greatest differences were observed in the contents of the compounds formed by enzymatic degradation of polyunsaturated fatty acids; the plum spirit made from pulped fruits (P8) contained significantly more 1-hexanol, *Z*-3-hexenol, 1-nonanol and hexanal than the plum spirit made from crushed fruits (P6).

By comparing the plum spirits made from the pulped mashes, it was observed that the addition of baker’s yeast led to several compositional changes. When compared to the plum spirits made from the spontaneously fermented pulped plum mashes (P8 vs. P7 and P9), the addition of yeast increased the contents of 1-propanol, ethyl octanoate, 1,1-diethoxy ethane, hexanoic acid, and octanoic acid. Conversely, it decreased ethyl propanoate and phenylethyl acetate. These changes are likely attributable to the different metabolic activities of the *Saccharomyces* strains present in the pulped mashes [11,49].

### 3.5. Odor Activity Values and Sensory Characteristics of Plum Spirits

Although present in high concentrations, some plum spirit components, due to high odor threshold (OT) values, have a limited impact on aroma. Low odor activity values (OAVs < 1) indicate a low or a slight contribution to aroma. An odor activity value (OAV) > 1 indicates that a compound contributes to the plum spirit aroma. However, components with an OAV between 0.2 and 1.0 are also considered to contribute to the aroma of the final product, but to a lesser extent [42,56]. Among the 39 analyzed compounds (Table S1, Supplementary Materials), the number of compounds with an OAV > 1 in the plum spirits from sound fruits was 16 for G1 and 15 for G2, and in the plum spirits from heat-damaged fruits, it was 16 (P2), 17 (P1, P3, P4, P6, P9), 19 (P5, P7) and 20 (P8). The number of compounds whose odor activity values were in the range 0.2 < OAV < 1 was six (G1 and G2) in the plum spirits produced from sound fruits and three (P8), four (P5, P6, P7), five (P9), seven (P2, P3, P4) and eight (P1) in the ones from heat-damaged fruits.

**Figure 3:**
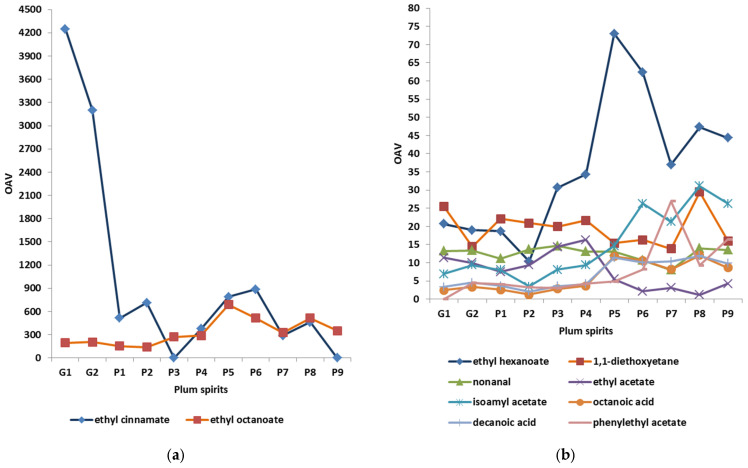

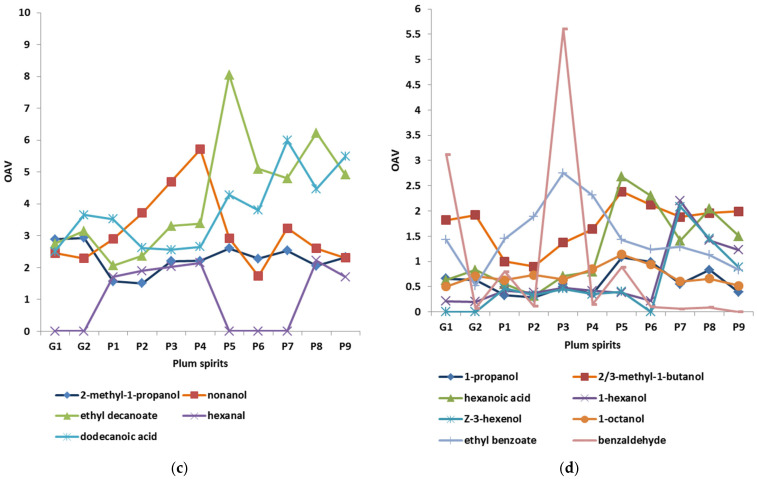
Odor activity value (OAV) profiles of plum spirits from sound fruits (G1–G2) and heat-damaged fruits (P1–P9): (**a**) compounds that had OAVs > 100 in at least one sample of plum spirit, (**b**) compounds that had OAVs > 10 in at least one sample, (**c**) compounds with an OAV between 1 and 10 in all plum spirit samples, and (**d**) compounds whose OAVs, depending on the sample, were either >1 or between 0.2 and 1.

In order to better visualize the data in Table S1 (Supplementary Materials), we classified the components of the plum spirits according to the OAVs into four groups (Figure 3):

(a) Compounds that had an OAV > 100 in at least one sample of plum spirit (ethyl cinnamate and ethyl octanoate);
(b) Compounds that had an OAV > 10 in at least one sample of plum spirit (nonanal, 1,1-diethoxy ethane; ethyl hexanoate, isoamyl acetate, phenylethyl acetate, octanoic acid, decanoic acid, ethyl acetate);
(c) Compounds with an OAV between 1 and 10 in all plum spirit samples (nonanol, hexanal, 2-methyl-1-propanoate, ethyl decanoate, dodecanoic acid);
(d) Compounds whose OAV, depending on the sample, was either >1 or between 0.2 and 1 (1-propanol, 2/3-methyl-1-butanol, hexanoic acid, 1-hexanol, *Z*-3-hexenol, 1-octanol, ethyl benzoate, benzaldehyde).

The other compounds had OAVs < 1 in all samples. The only compound with an OAV between 0.2 and 1 in all plum spirits was 1-butanol. The compounds with OAVs > 0.2 only in some samples were: 2-phenylethanol (G1, G2, P9), ethyl lactate (P3), propyl acetate (G2, P3), heptanal (P1, P2, P3, P4). 1-Heptanol, benzyl alcohol, ethyl propanoate, methyl acetate, acetaldehyde, furfural, and acetic acid could not contribute to aroma because they had OAVs < 0.2 in all plum spirits.

Velišek et al. [57] demonstrated that among 13 compounds responsible for the characteristic plum-like odor of plum spirits, benzaldehyde, ethyl cinnamate and nonanal were quantitatively most abundant. In our study, the highest OAVs for ethyl cinnamate were found in the plum spirits from sound fruits (4250 and 3200 in samples G1 and G2, respectively); the highest OAVs for benzaldehyde were found in two plum spirits from the mashes with stones, both from sound and heat-damaged fruits (3.12 and 5.61 in samples G1 and P3, respectively). The OAVs for nonanal in all plum spirits were between 10.00 and 15.00, except for sample P7. The abovementioned authors found that the following compounds also gave the characteristic plum-like note of plum spirits: hexanal, heptanal and phenylethyl acetate. These compounds were found in our samples.

**Figure 4:**
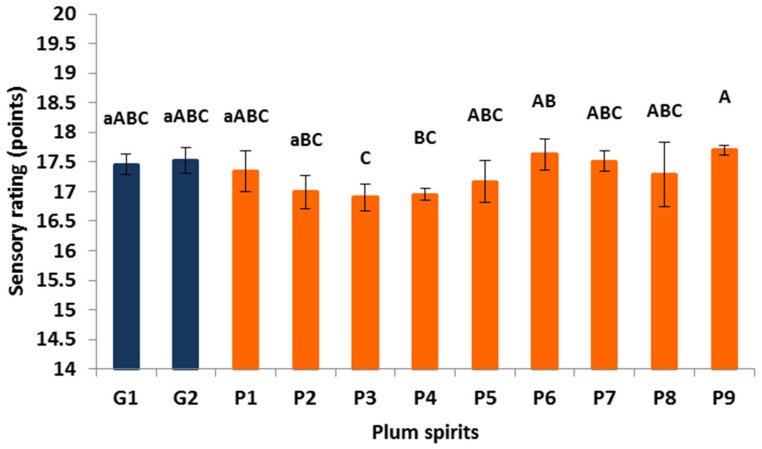
Sensory ratings of plum spirits produced from sound (G1–G2) and heat-damaged fruits (P1–P2). Different lowercase letters indicate statistically significant differences among the four samples (G1, G2, P1, and P2) produced from sound and heat-damaged fruits of ‘Čačanska Lepotica’ (Tukey’s test, *p* < 0.05). Different uppercase letters in the same row indicate statistically significant differences among all plum spirit samples (G1–G2 and P1–P9) (Tukey’s test, *p* < 0.05). Ratings of all plum spirits are shown as mean ± standard deviation.

The sensory ratings of all plum spirits were in the range of 16.90–17.70, and according to the Buxbaum scale, they were all classified within the quality category of products awarded a silver medal. However, there were subtle differences in the sensory ratings affected by the health status of the plums and the production method applied (Figure 4 and Table S2, Supplementary Materials).

The plum spirits from heat-damaged fruits obtained in the same way as those from sound fruits (comparison of G1 and P1, as well as G2 and P2) were rated lower. This was not statistically significant for the plums processed with stones (comparison of P1 and G1), but the differences in the sensory ratings were significant between the samples obtained from the stoneless plum mashes (comparison of P2 and G2) due to the appearance of a slightly pronounced ‘cooked plum’ note or a ‘note of overripe plum’ in the plum spirit produced from heat-damaged plums. It is possible that this undesirable ‘cooked plum’ note occurred in sample P2 due to a significantly higher OAV for 1-nonanol compared to sample G2 (OAVs were 3.71 and 2.29, respectively).

Because the sensory ratings of samples P1 and P2 were lower than those of samples G1 and G2, all modifications to the production method were carried out with the aim of improving the sensory properties of the plum spirit obtained from unsuitable raw materials. Distillation of the fermented mash, stored for 15 days after the end of fermentation, led to a further decrease in the sensory ratings (comparison of P3 and P1, as well as P4 and P2). In addition to a slight reduction in the finesse of the fruity aroma, caused by decreased OAVs of certain ethyl esters, these plum spirits had a more pronounced sour taste due to the increased total acid content. They also exhibited a stronger ‘cooked plum’ aroma, attributed to the highest OAVs of 1-nonanol found in samples P3 (4.70) and P4 (5.71). These plum spirits had OAVs for ethyl acetate that were almost two times higher than those of samples P1 and P2, which led to a pronounced ‘solvent-like’ note and a further reduction in the fine fruity character. In addition, the highest OAVs for benzaldehyde (5.61) and ethyl benzoate (2.75) were found in plum spirit P3, so it had a slightly more pronounced stone-like and cherry-stone notes [11] that were not compatible with the fruity character of plum spirits from this variety [6]. Plum spirits P3 and P4 were rated the lowest (16.90 and 16.95, respectively). The use of baker’s yeast in the fermentation of the crushed plum mash with stones did not significantly change the sensory rating of the plum spirit from heat-damaged fruits (comparison of samples P1 and P5). However, fermentation of the crushed plum mash without stones using baker’s yeast significantly increased the sensory rating of the plum spirit from 16.99 (sample P2) to 17.63 (sample P6). Such large differences in the ratings of P2 and P6 can be attributed to significantly lower OAVs for 1-nonanol and ethyl acetate and higher OAVs for certain esters with pronounced fruity notes in sample P6. Sample P6 had a concentration of total acids that was almost four times lower than that of sample P2, which significantly affected higher taste acceptability (P2 was rated 7.96 points for taste, and P6 was rated 8.45). The sensory rating of the plum spirits from the pulped plum mash without stones (17.51, 17.29 and 17.70 in P7, P8 and P9, respectively) differed due to the different types of fermentation (spontaneous fermentation or addition of baker’s yeast) and different pH values of the mash (native pH or corrected to pH 3.0). These differences in the processing methods led to the selection of microorganisms in the mash during alcoholic fermentation, and thus to a modification of the aromatic profile of plum spirits [11,58].

In general, several samples (G1, G2, P1, P5, P7 and P8) did not differ significantly in terms of sensory ratings, which were in the range of 17.17–17.53, although some of these samples had completely different aromatic profiles. Among them, the highest OAVs for ethyl cinnamate were found in the plum spirits from sound fruits (G1 and G2), so they had a pronounced plum aroma, and G2 also had a fruity-floral note (the highest OAVs for 2-methyl-1-propanol and 2-phenylethanol). Samples P5 and P8 had the highest OAVs for esters that give a fruity character (for ethyl octanoate, ethyl hexanoate, and ethyl decanoate in P5; for isoamyl acetate in P8) and also for fatty acids, certain alcohols or aldehydes (for hexanoic acid, 2/3-methyl-1-butanol, 1-propanol, and 1-octanol in P5; for 1,1-diethoxyethane, octanoic acid, decanoic acid, and hexanal in P8), which may slightly impair their harmony and pleasant sensory perception. In sample P1, it was observed that the OAV of any compound was not higher than in the other samples. Sample P7 had the highest OAVs for phenylethyl acetate, dodecanoic acid, 1-hexanol, and Z-3-hexenol.

The harmonious balance of individual compounds contributes much more to the complex and pleasant aromatic profile of a plum spirit than the dominance of some of them [59]. Our results are in accordance with previous findings: in the best-rated plum spirit, P9 (17.70), no OAVs of the analyzed compounds were higher than in the other samples. In the case of sample P9, the processing method that included pulping of plums (with simultaneous removal of the stones), lowering the natural pH to 3.0, and spontaneous fermentation enabled us to obtain a high-quality plum spirit, with a fresh, fruity–floral aroma, without the presence of undesirable sensory notes, even from heat-damaged fruits.

The four best plum spirits were rated more than 17.50 points: one produced from sound plums—G2 (17.53) and three from heat-damaged plums—P7 (17.51), P6 (17.63) and P9 (17.70). All of them were made from the stoneless mashes—two from crushed plums (G2 and P6) and two from pulped plums (P7 and P9). From these results, it is clear that if heat-damaged fruits are processed into plum spirit, with no possibility of pulping, it is necessary to add baker’s yeast to a mash of crushed plums without stones (P6). However, if a distillery has the capacity for fruit pulping, a high sensory rating can also be achieved using spontaneous fermentation (P7), which can be further enhanced by lowering the mash pH to 3.0 before fermentation (P9).

### 3.6. Principal Component Analysis (PCA)

**Figure 5:**
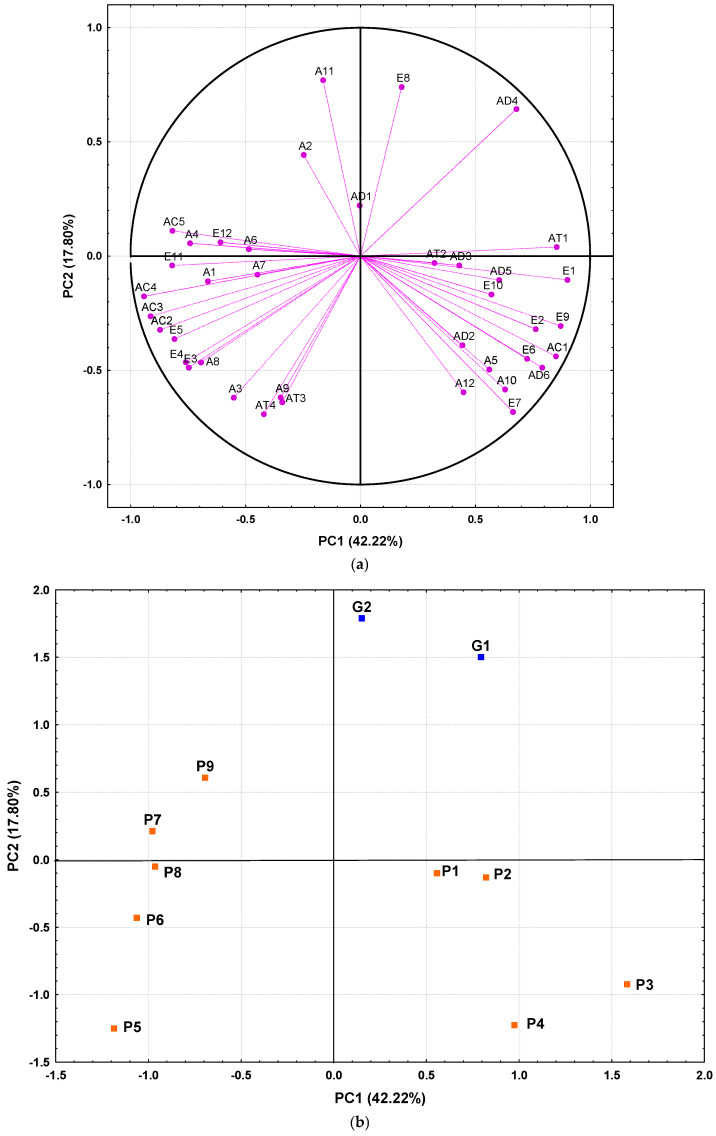
Principal component analysis (PCA) calculated on 39 volatile compounds. (**a**) Variable loadings. Codes of compounds are shown in Table 4. (**b**) Sample scores. Plum spirits obtained from sound fruits (G1–G2) and heat-damaged fruits (P1–P9) of ‘Čačanska Lepotica’.

PCA was applied to determine the possibility of grouping the plum spirits obtained from sound and heat-damaged fruits by the different production methods, based on the concentration of 39 volatiles (analyzed by GC-MS). The first two principal components explained 60.02% (42.22% and 17.80%, respectively) of the total variance (Figure 5).

The fruit health status, as well as the different processing methods, had a complex impact on the aromatic profile and sensory evaluations of the plum spirits. However, PCA enabled clear separation and grouping of the samples. The plum spirits obtained by spontaneous fermentation of crushed plums (with or without stones) (G1–G2 and P1–P4) contained significantly more esters (ethyl acetate, methyl acetate, ethyl lactate, ethyl propanoate), typical of bacterial activity in spontaneously fermented mashes, and were located on the positive side of PC1. Those located on the negative side of PC1 included the plum spirits obtained from heat-damaged plums, produced from the crushed plum mashes (with or without stones) with the addition of baker’s yeast, as well as from the pulped fruits regardless of the fermentation type and pH value of the mash (P5–P6 and P7–P9). They were characterized by higher contents of higher alcohols, fatty acids and esters.

Separation along the PC2 axis enabled further grouping of the samples. On the positive side of PC1, one separate group was formed of samples G1 and G2 (on the positive side of PC2), which contained the highest contents of ethyl cinnamate (characteristic plum odor), and the other group consisted of samples P1–P4 (on the negative side of PC2), which contained the most nonanol (cooked plum odor). On the negative side of PC1, one group (on the negative side of PC2) consisted of the samples obtained using baker’s yeast (P5, P6 and P8), and the second group (on the positive side of PC2) consisted of samples from the pulped heat-damaged fruit, spontaneously fermented (P7 and P9), which were ranked among the four best-rated plum spirits in terms of sensory quality.

## 4. Conclusions

The plum spirits obtained from heat-damaged fruits, regardless of the processing method, met the requirements of the legislation regarding the contents of harmful methanol and HCN, as well as the content of total volatile substances. However, the use of traditional processing methods (distillation of mash from crushed plums, which were spontaneously fermented), which are effective when sound fruits of this variety are used, was not effective when processing heat-damaged fruits. Plum spirits produced from such fruits lacked finesse, freshness and a pleasant varietal aroma; they had a ‘cooked plum’ aroma (samples P1 and P2) that was especially pronounced when the fermented mash was not distilled immediately after fermentation, but after 15 days of storage (samples P3 and P4). In order to improve the aromatic profile of plum spirits from heat-damaged fruits, it is necessary to modify the processing method. Good sensory quality of plum spirits produced from crushed heat-damaged fruits can be achieved by removing stones from the crushed plum mash and adding yeast (sample P6). By pulping plums, it is possible to significantly modify the chemical composition of plum spirits and thus, regardless of spontaneous fermentation, obtain plum spirits with a fruity character (samples P7 and P9), which is especially pronounced if the pH of pulped plums is lowered to 3.0 prior to alcoholic fermentation. Using a modified production method, distilleries can obtain plum spirits from heat-damaged plums with even better sensory quality than plum spirits produced from sound fruits of this variety using the traditional method.

## Figures and Tables

**Table 1:** Basic characteristics of plum fruits for plum spirit production.

Fruit Characteristics	Heat-Damaged	Sound
	fruits	fruits
Locality	Preljina (P)	Gledjica (G)
Altitude (m)	390	790
GPS coordinates	43°92′41.61″ N	43°43′80.49″ N
	20°44′84.94″ E	20°21′48.20″ E
Harvest date	9 August 2007	17 August 2007
Fruit weight (g)	38.83	29.94
Stone weight (g)	1.65	1.30
Stone ratio (%)	4.25	4.34
Soluble solids (%)	15.70	16.00
Total sugars (%)	11.70	12.45
Inverted sugars (%)	7.35	6.48
Sucrose (%)	4.13	5.67
Total acids (%)	0.70	1.18
pH	3.79	3.49
Soluble solids/acids ratio	22.43	13.56
Sugars/acids ratio	16.71	10.55
Pectinic acid (mg AGA/100 g fruits)	190.10	171.30
Pectic acid (mg AGA/100 g fruits)	40.30	20.25
Protopectin (mg AGA/100 g fruits)	540.40	439.90
Total pectin (mg AGA/100 g fruits)	770.80	631.45
Pectinic acid/protopectin ratio	0.35	0.39
Sugars/pectin ratio ^1^	15.19	19.76

AGA: anhydro-galacturonic acid. ^1^: For the calculation of the sugars/pectin ratio, the concentrations of total pectin (shown in Table 1) are expressed as a percentage (%) on a fresh weight basis instead of mg AGA/100 g fruits.

**Table 2:** Modifications to plum spirit production methods.

Plum Spirit Samples	G1	G2	P1	P2	P3	P4	P5	P6	P7	P8	P9
Locality	G	G	P	P	P	P	P	P	P	P	P
Stones in the mash	w	wo	w	wo	w	wo	w	wo	wo	wo	wo
Fruit mashing	c	c	c	c	c	c	c	c	p	p	p
Mash pH value	n	n	n	n	n	n	n	n	n	n	a
Alcoholic fermentation	s	s	s	s	s	s	y	y	s	y	s
Fermented mash storage time (days)	0	0	0	0	15	15	0	0	0	0	0

G: Gledjica locality; P: Preljina locality; w: with stones in the mash; wo: without stones in the mash; c: crushing; p: pulping; n: native pH; a: pH adjusted to pH 3; s: spontaneous alcoholic fermentation; y: added yeast; 0: 0 days; 15: 15 days.

**Table 3:** The ratios of higher alcohols in plum spirits obtained from sound fruits (G1–G2) and from heat-damaged fruits (P1–P9).

Plum Spirit Sample	Ratio		
	IA/IB	IA/1P	IB/1P
G1	1.87	1.22	0.66
G2	1.77	1.13	0.64
P1	1.61	1.00	0.62
P2	1.50	1.00	0.67
P3	1.68	1.04	0.62
P4	1.98	1.74	0.88
P5	2.51	0.86	0.34
P6	2.51	0.82	0.33
P7	1.93	1.18	0.61
P8	2.62	0.91	0.35
P9	2.34	1.92	0.82

IA: isoamyl alcohol (sum of 2- and 3-methyl-1-butanol); IB: isobutanol (syn. 2-methyl-1-propanol); 1P: 1-propanol.

**Table 4:** Volatile aroma compounds in plum spirits (analyzed by GC-MS).

Compounds (mg/L)	Code	Plum Spirits										
		G1	G2	P1	P2	P3	P4	P5	P6	P7	P8	P9
Alcohols												
1-Propanol	A1	35.87 ± 5.38 ^aBC^	34.12 ± 4.27 ^aBC^	17.59 ± 1.45 ^bD^	15.65 ± 1.10 ^bD^	26.37 ± 3.79 ^CD^	18.65 ± 1.01 ^D^	58.39 ± 9.17 ^A^	53.40 ± 3.18 ^A^	29.71 ± 2.91 ^CD^	44.79 ± 8.99 ^AB^	21.27 ± 3.92 ^CD^
2-Methyl-1-propanol	A2	81.67 ± 12.38 ^aAB^	82.79 ± 8.40 ^aA^	44.41 ± 3.42 ^bC^	42.70 ± 3.20 ^bC^	62.36 ± 6.03 ^ABC^	62.52 ± 2.50 ^ABC^	73.68 ± 10.22 ^AB^	64.58 ± 1.82 ^ABC^	71.73 ± 6.80 ^AB^	58.11 ± 10.52 ^BC^	65.79 ± 11.21 ^ABC^
1-Butanol	A3	0.89 ± 0.16 ^aCD^	0.62 ± 0.06 ^aD^	0.83 ± 0.09 ^aCD^	0.78 ± 0.03 ^aCD^	1.00 ± 0.10 ^BC^	1.25 ± 0.04 ^B^	2.27 ± 0.30 ^A^	1.96 ± 0.09 ^A^	1.03 ± 0.05 ^BC^	1.10 ± 0.02 ^BC^	0.97 ± 0.18 ^BCD^
2/3-Methyl-1-butanol	A4	326.15 ± 53.00 ^aABC^	343.86 ± 27.78 ^aABC^	179.62 ± 13.63 ^bD^	161.24 ± 14.76 ^bD^	244.93 ± 15.53 ^CD^	293.10 ± 10.45 ^BC^	425.68 ± 53.62 ^A^	380.11 ± 6.57 ^AB^	337.37 ± 32.58 ^ABC^	349.97 ± 59.07 ^ABC^	355.82 ± 56.89 ^AB^
1-Pentanol	A5	ND ^aD^	ND ^aD^	ND ^aD^	ND ^aD^	1.27 ± 0.01 ^A^	0.94 ± 0.01 ^B^	ND ^D^	ND ^D^	0.41 ± 0.00 ^C^	ND ^D^	ND ^D^
1-Hexanol	A6	1.15 ± 0.22 ^bC^	1.08 ± 0.07 ^bC^	2.26 ± 0.17 ^aC^	2.02 ± 0.23 ^aC^	2.60 ± 0.13 ^C^	2.24 ± 0.07 ^C^	2.06 ± 0.23 ^C^	1.16 ± 0.04 ^C^	11.83 ± 1.14 ^A^	7.62 ± 1.21 ^B^	6.59 ± 0.96 ^B^
*Z*-3-Hexenol	A7	ND ^cE^	ND ^cE^	0.59 ± 0.07 ^aD^	0.41 ± 0.02 ^bD^	0.56 ± 0.05 ^D^	0.44 ± 0.01 ^D^	0.50 ± 0.05 ^D^	ND ^E^	2.62 ± 0.19 ^A^	1.82 ± 0.28 ^B^	1.11 ± 0.20 ^C^
1-Heptanol	A8	ND ^bD^	0.29 ± 0.02 ^aC^	0.29 ± 0.01 ^aC^	0.33 ± 0.05 ^aC^	ND ^D^	0.47 ± 0.02 ^B^	0.66 ± 0.08 ^A^	0.50 ± 0.03 ^B^	0.40 ± 0.03 ^BC^	0.39 ± 0.04 ^BC^	0.32 ± 0.07 ^C^
1-Octanol	A9	0.55 ± 0.05 ^bF^	0.78 ± 0.03 ^aCDE^	0.69 ± 0.04 ^abDEF^	0.80 ± 0.11 ^aCD^	0.70 ± 0.02 ^DEF^	0.93 ± 0.07 ^BC^	1.25 ± 0.05 ^A^	1.03 ± 0.02 ^AB^	0.66 ± 0.06 ^DEF^	0.73 ± 0.15 ^CDEF^	0.57 ± 0.13 ^EF^
1-Nonanol	A10	2.45 ± 0.40 ^bDEF^	2.29 ± 0.09 ^bEF^	2.90 ± 0.25 ^abCDE^	3.71 ± 0.43 ^aC^	4.70 ± 0.28 ^B^	5.71 ± 0.27 ^A^	2.92 ± 0.29 ^CDE^	1.73 ± 0.02 ^F^	3.24 ± 0.30 ^CD^	2.60 ± 0.40 ^DE^	2.31 ± 0.34 ^EF^
2-Phenylethanol	A11	5.80 ± 1.01 ^bB^	7.60 ± 0.51 ^aA^	4.29 ± 0.33 ^bcB^	4.06 ± 0.46 ^cB^	4.77 ± 0.10 ^B^	4.38 ± 0.23 ^B^	4.93 ± 0.56 ^B^	4.78 ± 0.04 ^B^	5.54 ± 0.61 ^B^	4.67 ± 0.73 ^B^	5.77 ± 0.95 ^B^
Benzyl alcohol	A12	1.57 ± 0.25 ^aC^	0.43 ± 0.02 ^bD^	1.37 ± 0.08 ^aC^	1.26 ± 0.12 ^aC^	3.92 ± 0.15 ^A^	1.63 ± 0.10 ^C^	2.82 ± 0.29 ^B^	1.61 ± 0.04 ^C^	0.22 ± 0.02 ^DE^	0.33 ± 0.00 ^DE^	ND ^E^
**Esters**												
Ethyl acetate	E1	372.02 ± 63.51 ^aBC^	324.04 ± 28.39 ^abCD^	245.55 ± 19.13 ^bDE^	304.62 ± 22.62 ^abCD^	467.74 ± 84.07 ^AB^	532.30 ± 18.05 ^A^	178.65 ± 26.91 ^EF^	69.86 ± 3.37 ^G^	102.86 ± 10.39 ^FG^	39.21 ± 6.39 ^G^	138.45 ± 18.16 ^FG^
Ethyl propanoate	E2	0.61 ± 0.10 ^aBC^	0.52 ± 0.06 ^aCD^	0.48 ± 0.03 ^aCD^	0.46 ± 0.04 ^aCD^	1.09 ± 0.12 ^A^	0.80 ± 0.03 ^B^	0.54 ± 0.08 ^CD^	0.36 ± 0.02 ^DE^	0.41 ± 0.03 ^CD^	0.20 ± 0.03 ^E^	0.41 ± 0.09 ^D^
Ethyl hexanoate	E3	0.62 ± 0.11 ^aEF^	0.57 ± 0.04 ^aEF^	0.56 ± 0.04 ^aEF^	0.31 ± 0.01 ^bF^	0.92 ± 0.08 ^DE^	1.03 ± 0.05 ^CDE^	2.19 ± 0.35 ^A^	1.87 ± 0.25 ^AB^	1.11 ± 0.10 ^CD^	1.42 ± 0.15 ^BC^	1.33 ± 0.29 ^CD^
Ethyl octanoate	E4	2.51 ± 0.45 ^aDE^	2.64 ± 0.12 ^aDE^	1.98 ± 0.15 ^abE^	1.78 ± 0.18 ^bE^	3.51 ± 0.21 ^CD^	3.71 ± 0.22 ^CD^	8.89 ± 0.95 ^A^	6.64 ± 0.14 ^B^	4.22 ± 0.46 ^C^	6.62 ± 0.95 ^B^	4.54 ± 0.73 ^C^
Ethyl decanoate	E5	3.08 ± 0.51 ^abE^	3.53 ± 0.18 ^aE^	2.31 ± 0.17 ^bE^	2.65 ± 0.35 ^bE^	3.71 ± 0.16 ^DE^	3.79 ± 0.21 ^CDE^	9.02 ± 1.01 ^A^	5.71 ± 0.10 ^B^	5.38 ± 0.59 ^BCD^	6.98 ± 1.04 ^B^	5.50 ± 0.85 ^BC^
Ethyl lactate	E6	4.64 ± 0.76 ^bDE^	3.31 ± 0.30 ^bEF^	19.8 ± 1.58 ^aB^	5.68 ± 0.54 ^bD^	28.63 ± 1.01 ^A^	15.23 ± 0.55 ^C^	5.06 ± 0.57 ^DE^	2.10 ± 0.06 ^F^	3.41 ± 0.37 ^EF^	2.41 ± 0.38 ^F^	3.81 ± 0.67 ^DEF^
Ethyl benzoate	E7	2.05 ± 0.30 ^bC^	0.74 ± 0.02 ^cE^	2.08 ± 0.17 ^bC^	2.70 ± 0.33 ^aB^	3.93 ± 0.26 ^A^	3.30 ± 0.12 ^AB^	2.05 ± 0.15 ^C^	1.78 ± 0.06 ^CD^	1.85 ± 0.24 ^C^	1.61 ± 0.26 ^CD^	1.19 ± 0.19 ^DE^
Ethyl cinnamate	E8	3.40 ± 0.90 ^aA^	2.56 ± 0.17 ^aA^	0.41 ± 0.02 ^bB^	0.57 ± 0.04 ^bB^	ND ^B^	0.30 ± 0.01 ^B^	0.63 ± 0.10 ^B^	0.71 ± 0.05 ^B^	0.23 ± 0.00 ^B^	0.37 ± 0.00 ^B^	ND ^B^
Methyl acetate	E9	2.90 ± 0.54 ^aB^	1.47 ± 0.20 ^bBCD^	2.15 ± 0.15 ^abBC^	2.25 ± 0.15 ^abBC^	5.46 ± 1.63 ^A^	6.47 ± 0.45 ^A^	0.42 ± 0.04 ^D^	0.15 ± 0.02 ^D^	0.39 ± 0.07 ^D^	0.14 ± 0.03 ^D^	0.77 ± 0.15 ^CD^
Propyl acetate	E10	0.74 ± 0.11 ^bCDE^	0.98 ± 0.09 ^aAB^	0.49 ± 0.01 ^cFG^	0.52 ± 0.06 ^cEFG^	1.16 ± 0.11 ^A^	0.91 ± 0.06 ^BC^	0.80 ± 0.09 ^BCD^	0.67 ± 0.01 ^DEF^	0.35 ± 0.05 ^G^	0.34 ± 0.05 ^G^	0.37 ± 0.07 ^G^
Isoamyl acetate	E11	1.70 ± 0.26 ^bD^	2.30 ± 0.14 ^aCD^	1.96 ± 0.14 ^abD^	0.86 ± 0.06 ^cD^	1.99 ± 0.11 ^D^	2.29 ± 0.13 ^CD^	3.59 ± 0.48 ^C^	6.43 ± 0.18 ^AB^	5.21 ± 0.42 ^B^	7.62 ± 1.19 ^A^	6.44 ± 0.94 ^AB^
Phenylethyl acetate	E12	ND ^cF^	0.48 ± 0.02 ^aDE^	0.44 ± 0.02 ^aE^	0.35 ± 0.04 ^bEF^	0.33 ± 0.00 ^EF^	0.46 ± 0.02 ^DE^	0.52 ± 0.05 ^DE^	0.88 ± 0.02 ^CD^	2.91 ± 0.32 ^A^	1.06 ± 0.10 ^C^	1.78 ± 0.31 ^B^
**Aldehydes**												
Acetaldehyde	AD1	0.88 ± 0.09 ^aB^	0.49 ± 0.14 ^aB^	0.92 ± 0.03 ^aB^	0.98 ± 0.60 ^aAB^	0.66 ± 0.20 ^B^	0.77 ± 0.20 ^B^	0.50 ± 0.09 ^B^	0.37 ± 0.10 ^B^	0.85 ± 0.13 ^B^	0.91 ± 0.17 ^B^	1.71 ± 0.47 ^A^
Hexanal	AD2	ND ^bC^	ND ^bC^	0.27 ± 0.00 ^aB^	0.30 ± 0.03 ^aAB^	0.32 ± 0.01 ^AB^	0.34 ± 0.00 ^A^	ND ^C^	ND ^C^	ND ^C^	0.35 ± 0.02 ^A^	0.27 ± 0.06 ^B^
Nonanal	AD3	1.62 ± 0.40 ^aA^	1.63 ± 0.07 ^aA^	1.36 ± 0.32 ^aA^	1.67 ± 0.30 ^aA^	1.79 ± 0.39 ^A^	1.60 ± 0.32 ^A^	1.60 ± 0.73 ^A^	1.31 ± 0.47 ^A^	0.98 ± 0.18 ^A^	1.71 ± 0.36 ^A^	1.65 ± 0.26 ^A^
Furfural	AD4	6.54 ± 1.25 ^aA^	5.22 ± 0.23 ^abAB^	2.31 ± 0.23 ^cDEFG^	4.05 ± 0.50 ^bcBC^	4.24 ± 0.28 ^BC^	3.44 ± 0.28 ^CD^	1.17 ± 0.22 ^G^	1.37 ± 0.13 ^FG^	2.63 ± 0.16 ^DEF^	1.94 ± 0.32 ^EFG^	3.25 ± 0.50 ^CDE^
Benzaldehyde	AD5	13.11 ± 2.64 ^aB^	0.30 ± 0.02 ^bE^	3.38 ± 0.23 ^bCD^	0.48 ± 0.10 ^bE^	23.57 ± 1.82 ^A^	0.61 ± 0.04 ^DE^	3.75 ± 0.39 ^C^	0.42 ± 0.03 ^E^	0.27 ± 0.03 ^E^	0.37 ± 0.05 ^E^	ND ^E^
Heptanal	AD6	ND ^cD^	ND ^cD^	0.20 ± 0.01 ^bC^	0.27 ± 0.00 ^aA^	0.25 ± 0.00 ^B^	0.26 ± 0.00 ^AB^	ND ^D^	ND ^D^	ND ^D^	ND ^D^	ND ^D^
**Acetals**												
1,1-Ethoxy, methoxyetane	AT1	0.49 ± 0.06 ^aA^	0.19 ± 0.02 ^bCD^	0.34 ± 0.04 ^abABC^	0.30 ± 0.09 ^bBCD^	0.51 ± 0.04 ^A^	0.42 ± 0.12 ^AB^	ND ^E^	0.14 ± 0.00 ^DE^	0.16 ± 0.02 ^DE^	0.30 ± 0.07 ^BCD^	0.17 ± 0.04 ^CDE^
1,Diethoxy- etane	AT2	18.37 ± 2.37 ^aAB^	10.37 ± 0.76 ^bCD^	15.90 ± 1.74 ^aABC^	15.03 ± 2.71 ^abBCD^	14.35 ± 1.32 ^BCD^	15.59 ± 2.27 ^ABCD^	11.05 ± 0.94 ^CD^	11.71 ± 0.77 ^CD^	9.94 ± 2.83 ^D^	21.16 ± 3.05 ^A^	11.52 ± 1.87 ^CD^
1,1-Diethoxy- hexane	AT3	0.17 ± 0.02 ^bCD^	0.12 ± 0.03 ^bD^	0.50 ± 0.10 ^aAB^	0.51 ± 0.12 ^aAB^	0.26 ± 0.03 ^BCD^	0.43 ± 0.10 ^ABC^	0.52 ± 0.03 ^AB^	0.39 ± 0.09 ^ABC^	0.55 ± 0.17 ^A^	0.37 ± 0.13 ^ABCD^	0.35 ± 0.05 ^ABCD^
1,1-Diethoxy- nonane	AT4	1.31 ± 0.13 ^bCD^	0.91 ± 0.05 ^bD^	3.11 ± 0.65 ^aAB^	3.14 ± 0.73 ^aAB^	1.67 ± 0.39 ^BCD^	3.57 ± 0.32 ^A^	3.48 ± 0.87 ^A^	3.36 ± 0.91 ^AB^	3.48 ± 0.76 ^AB^	2.66 ± 0.63 ^ABCD^	2.81 ± 0.67 ^ABC^
**Acids**												
Acetic acid	AC1	3.27 ± 0.41 ^aBC^	1.06 ± 0.14 ^bEF^	2.75 ± 0.42 ^aCD^	4.03 ± 0.76 ^aB^	5.93 ± 0.38 ^A^	6.34 ± 0.23 ^A^	1.91 ± 0.11 ^DE^	0.71 ± 0.06 ^F^	1.32 ± 0.43 ^EF^	0.68 ± 0.15 ^F^	1.64 ± 0.37 ^EF^
Hexanoic acid	AC2	1.60 ± 0.27 ^bD^	2.09 ± 0.10 ^aD^	1.38 ± 0.07 ^bD^	0.80 ± 0.10 ^cD^	1.80 ± 0.10 ^D^	2.02 ± 0.12 ^D^	6.75 ± 0.73 ^A^	5.80 ± 0.13 ^AB^	3.56 ± 0.42 ^C^	5.14 ± 0.93 ^B^	3.79 ± 0.72 ^C^
Octanoic acid	AC3	6.45 ± 0.84 ^bC^	9.00 ± 0.33 ^aC^	6.96 ± 0.64 ^bC^	3.47 ± 0.43 ^cC^	7.58 ± 0.35 ^C^	9.75 ± 0.42 ^C^	31.80 ± 4.13 ^A^	28.68 ± 0.81 ^AB^	22.04 ± 2.73 ^B^	32.47 ± 4.87 ^A^	23.28 ± 4.10 ^B^
Decanoic acid	AC4	9.33 ± 1.67 ^bB^	12.78 ± 1.13 ^aB^	10.24 ± 0.84 ^abB^	5.83 ± 0.83 ^cB^	9.38 ± 0.80 ^B^	11.59 ± 0.62 ^B^	31.84 ± 4.10 ^A^	28.43 ± 0.23 ^A^	28.96 ± 3.84 ^A^	33.42 ± 5.08 ^A^	27.53 ± 4.63 ^A^
Dodecanoic acid	AC5	2.51 ± 0.60 ^cE^	3.65 ± 0.30 ^aCDE^	3.52 ± 0.30 ^abCDE^	2.62 ± 0.22 ^bcDE^	2.56 ± 0.24 ^DE^	2.65 ± 0.21 ^DE^	4.28 ± 0.74 ^ABCD^	3.80 ± 0.12 ^BCDE^	5.99 ± 0.88 ^A^	4.47 ± 0.62 ^ABC^	5.49 ± 1.29 ^AB^

Note: Different lowercase letters in the same row indicate statistically significant differences among the four samples (G1, G2, P1, and P2) produced from sound and heat-damaged fruits of ‘Čačanska Lepotica’ (Tukey’s test, *p* < 0.05); Different uppercase letters in the same row indicate statistically significant differences among all plum spirit samples (G1–G2 and P1–P9) (Tukey’s test, *p* < 0.05), ND—not detected. Concentrations of all compounds are shown as the mean of three replicates ± standard deviation.

## Data Availability

The original contributions presented in this study are included in the article/Supplementary Material. Further inquiries can be directed to the corresponding authors.

## Supplementary Materials

The following supporting information can be downloaded at https://www.mdpi.com/article/10.3390/foods15111855/s1: Figure S1: Key climate variables in July over a 25-year period (2000–2024) for the Čačak region (the Preljina locality); Figure S2: Key climate variables in August over a 25-year period (2000–2024) for the Čačak region (the Preljina locality); Figure S3: Maximum daily air temperatures in July and August 2007 (the Preljina locality); Figure S4: Kinetics of alcoholic fermentation of plum mashes from the ‘Čačanska Lepotica’ variety; Figure S5: Content of components prescribed by EU regulation in plum spirits produced from sound plums, the Gleđica locality (G1–G2), and from heat-damaged plums, the Preljina locality (P1–P9); Figure S6: Content of components prescribed by old Serbian regulation in plum spirits produced from sound plums, the Gleđica locality (G1–G2), and from heat-damaged plums, the Preljina locality (P1–P9); Figure S7: Profiles of major volatile components in plum spirits produced from sound plums, the Gledjica locality (G1–G2), and from heat-damaged plums, the Preljina locality (P1–P9) analyzed by GC-FID; Table S1: Odor activity values (OAVs) of volatile compounds in plum spirits obtained from sound fruits (G1–G2) and heat-damaged fruits (P1–P9) of ‘Čačanska Lepotica’; Table S2: Sensory ratings of plum spirits produced from sound (G1–G2) and heat-damaged fruits (P1–P2).
