# Supplementary material for: Heat-Damaged Fruits of ‘Čačanska Lepotica’ as a Raw Material for Plum Spirit Production: Possibilities for Improving Product Quality
Source: Foods. 2026 May 24;15(11):1855. doi: 10.3390/foods15111855 (PMC13256623; doi:10.3390/foods15111855)
Supplement: Supplementary file 1 [file foods-15-01855-s001.zip › foods-4225827-supplementary.pdf]

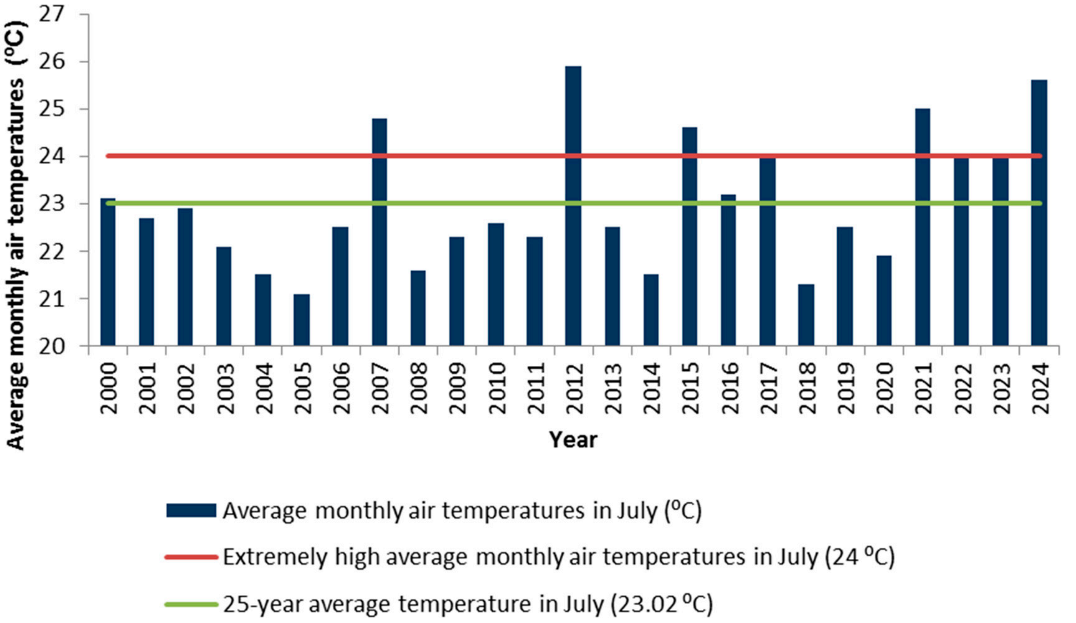
Average monthly air temperatures (°C)
27
26
25
24
23
22
21
20
2000 2001 2002 2003 2004 2005 2006 2007 2008 2009 2010 2011 2012 2013 2014 2015 2016 2017 2018 2019 2020 2021 2022 2023 2024
Year
Average monthly air temperatures in July (°C)
Extremely high average monthly air temperatures in July (24 °C)
25-year average temperature in July (23.02 °C)

(a1)

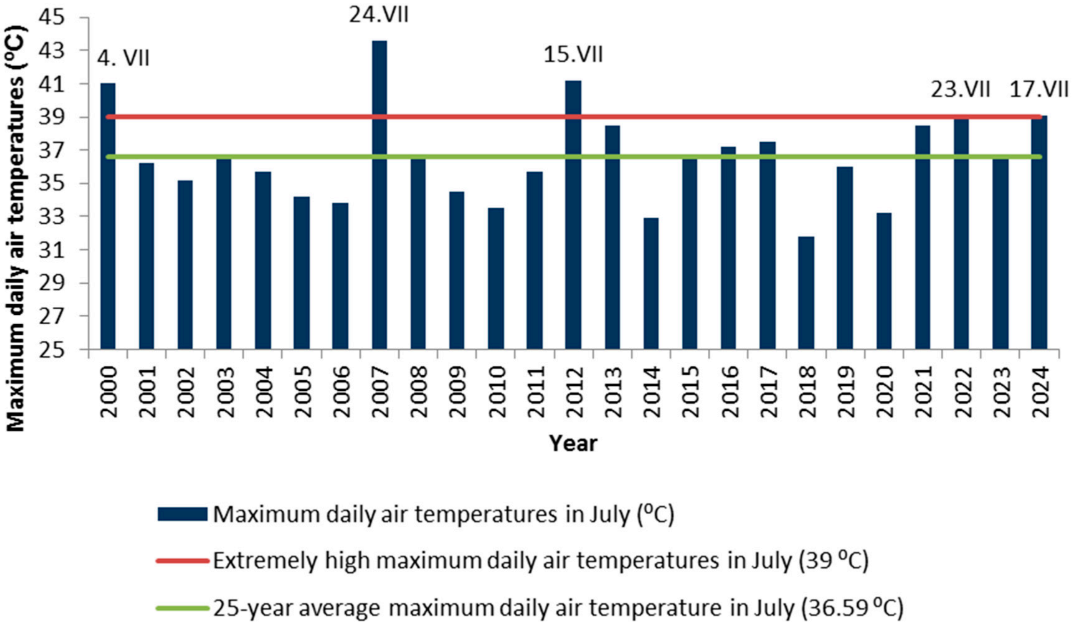
Maximum daily air temperatures (°C)
45
43
41
39
37
35
33
31
29
27
25
4. VII
24.VII
15.VII
23.VII
17.VII
2000 2001 2002 2003 2004 2005 2006 2007 2008 2009 2010 2011 2012 2013 2014 2015 2016 2017 2018 2019 2020 2021 2022 2023 2024
Year
Maximum daily air temperatures in July (°C)
Extremely high maximum daily air temperatures in July (39 °C)
25-year average maximum daily air temperature in July (36.59 °C)

(a2)

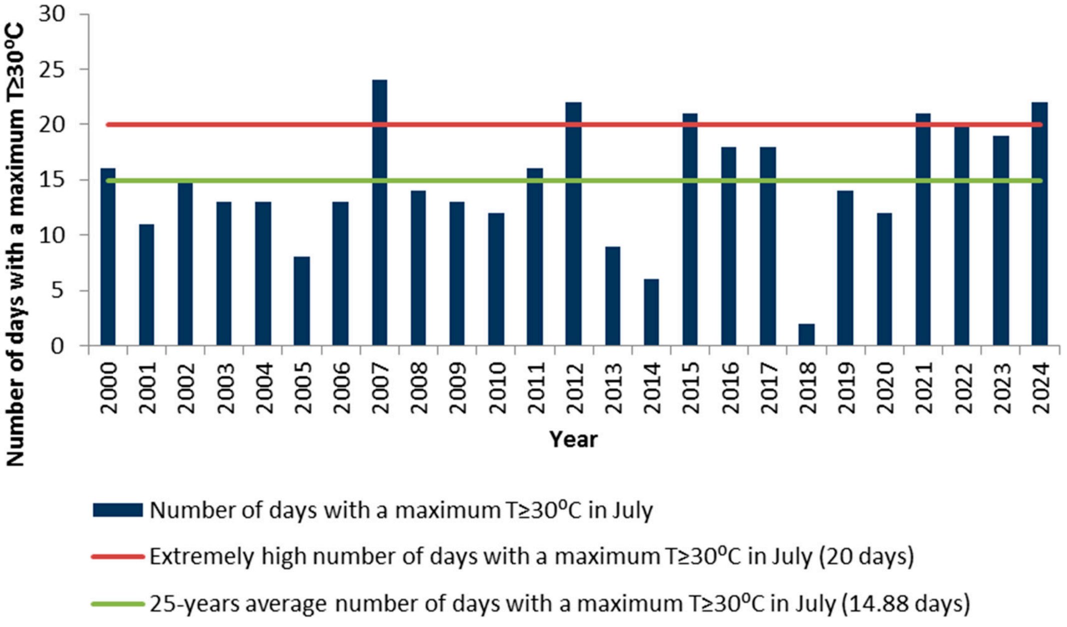

(a3)

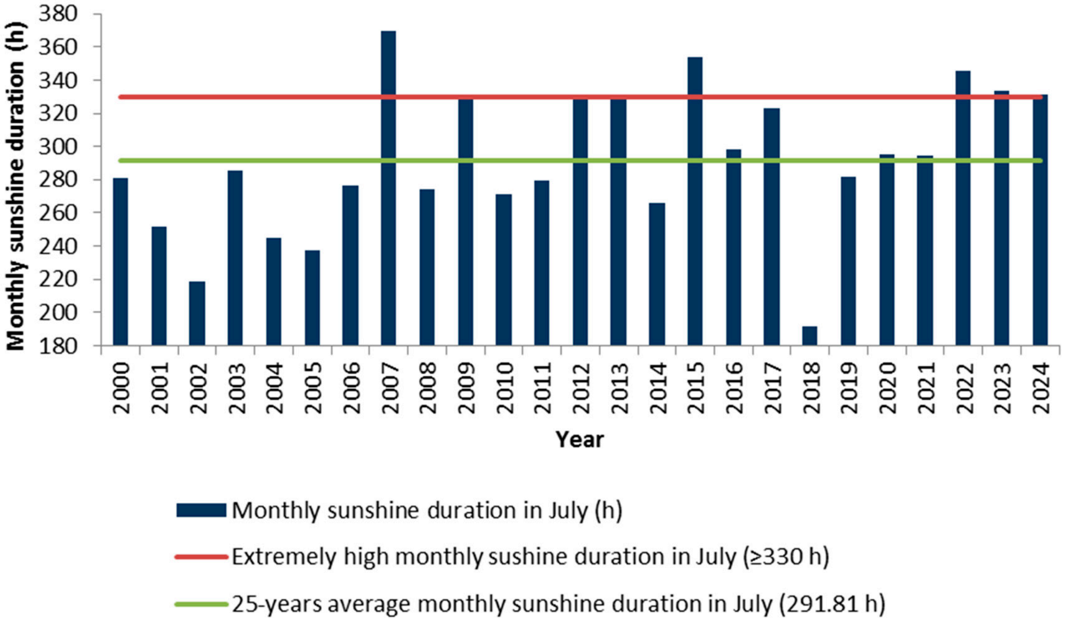

(a4)

Figure S1. Key climate variables in July over a 25-year period (2000–2024) for the Čačak region (the Preljina locality). a1) Average monthly air temperatures, a2) Maximum daily air temperatures, a3) Number of days with a maximum T ≥ 30°C, a4) Monthly sunshine duration.

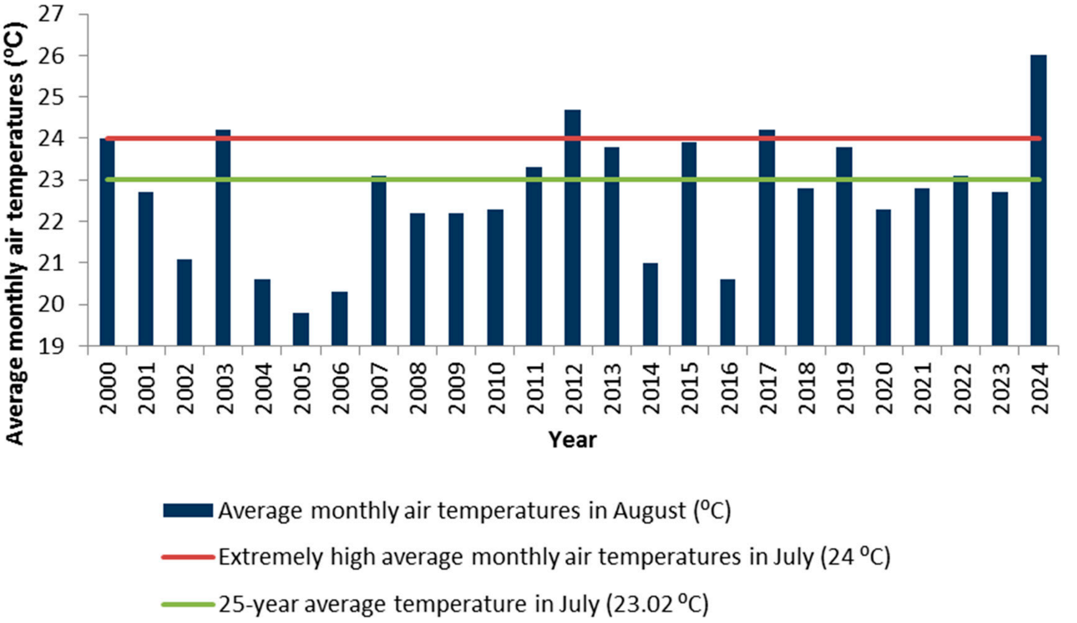
Average monthly air temperatures (°C)
27
26
25
24
23
22
21
20
19
2000
2001
2002
2003
2004
2005
2006
2007
2008
2009
2010
2011
2012
2013
2014
2015
2016
2017
2018
2019
2020
2021
2022
2023
2024
Year
Average monthly air temperatures in August (°C)
Extremely high average monthly air temperatures in July (24 °C)
25-year average temperature in July (23.02 °C)

(a1)

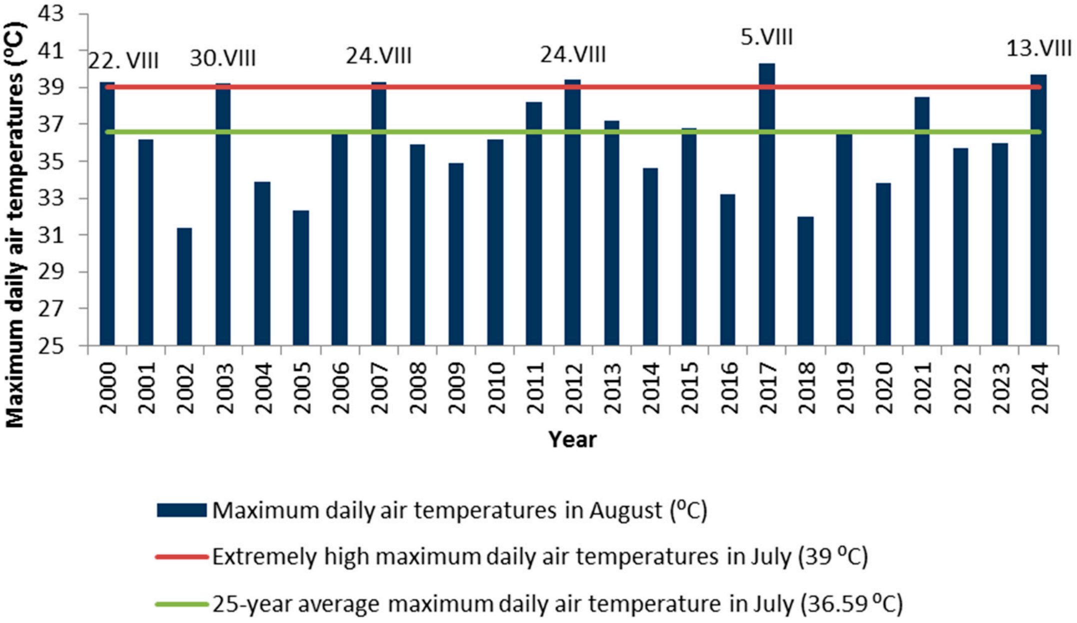
Maximum daily air temperatures (°C)
43
41
39
37
35
33
31
29
27
25
22. VIII
30.VIII
24.VIII
24.VIII
5.VIII
13.VIII
2000
2001
2002
2003
2004
2005
2006
2007
2008
2009
2010
2011
2012
2013
2014
2015
2016
2017
2018
2019
2020
2021
2022
2023
2024
Year
Maximum daily air temperatures in August (°C)
Extremely high maximum daily air temperatures in July (39 °C)
25-year average maximum daily air temperature in July (36.59 °C)

(a2)

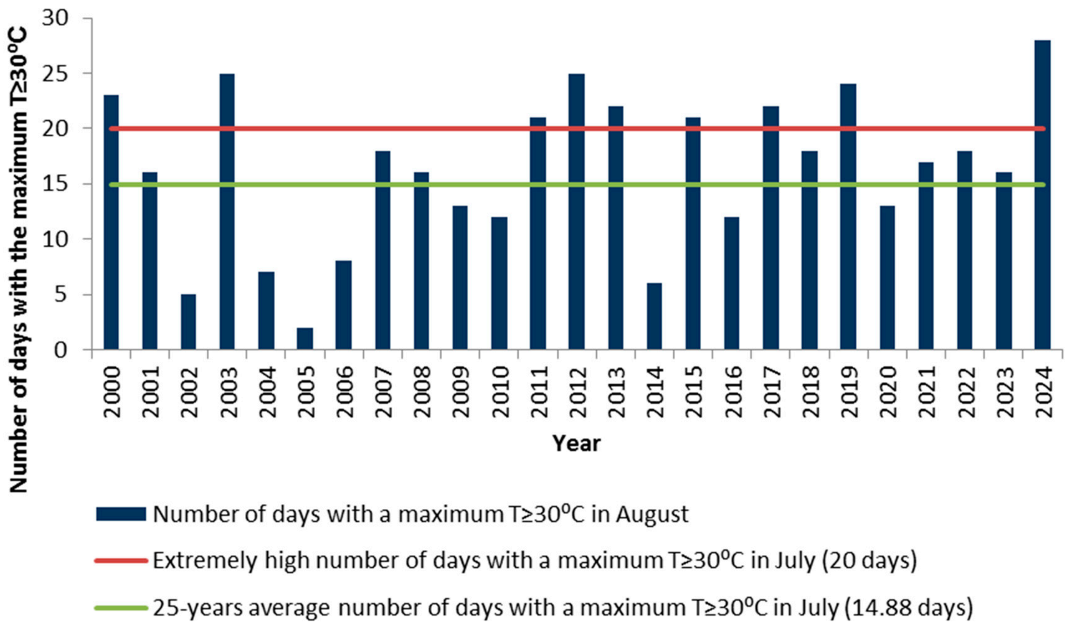

(a3)

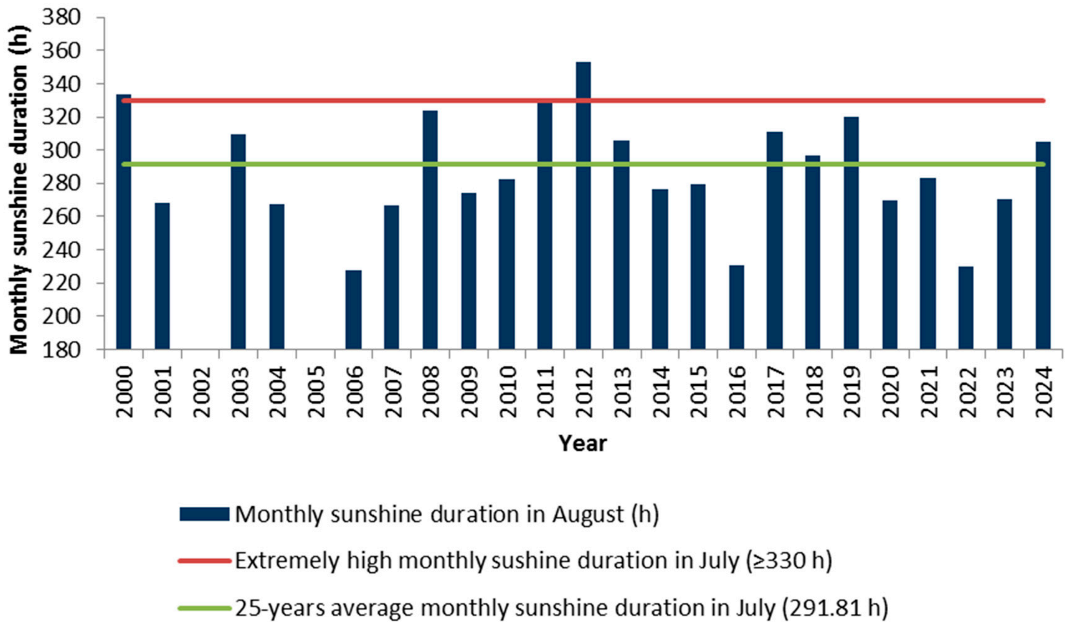

(a4)

Figure S2. Key climate variables in August over a 25-year period (2000–2024) for the Čačak region (the Preljina locality). a1) Average monthly air temperatures, a2) Maximum daily air temperatures, a3) Number of days with a maximum T ≥ 30°C, a4) Monthly sunshine duration.

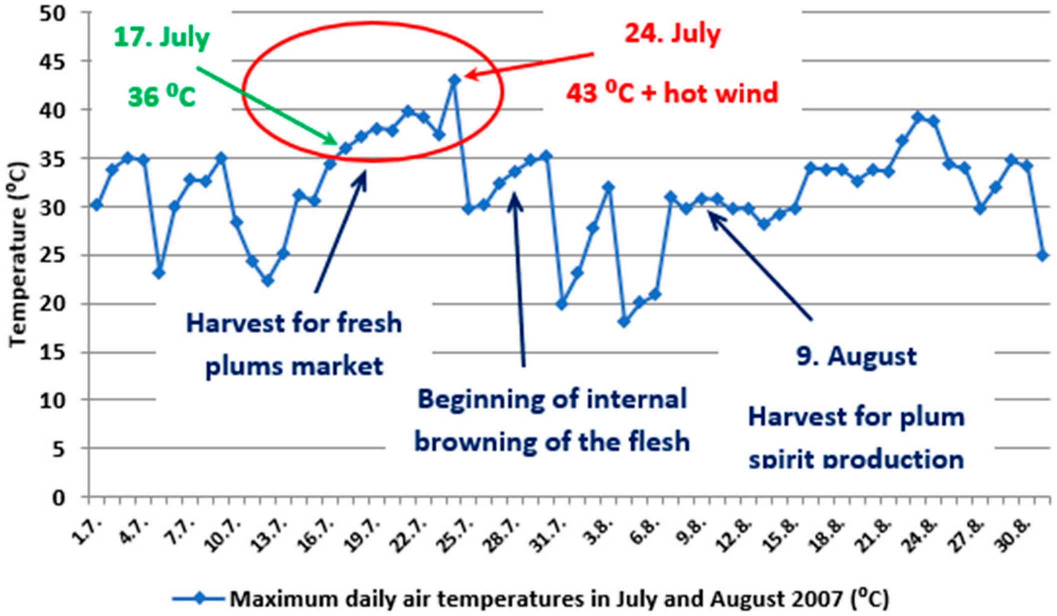

Figure S3. Maximum daily air temperatures in July and August 2007 (the Preljina locality). Red circle represents heat wave period.

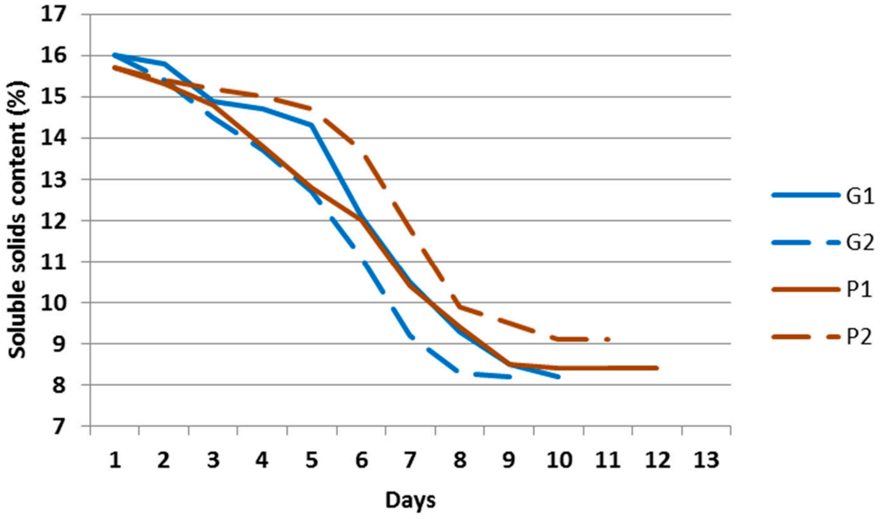

(a)

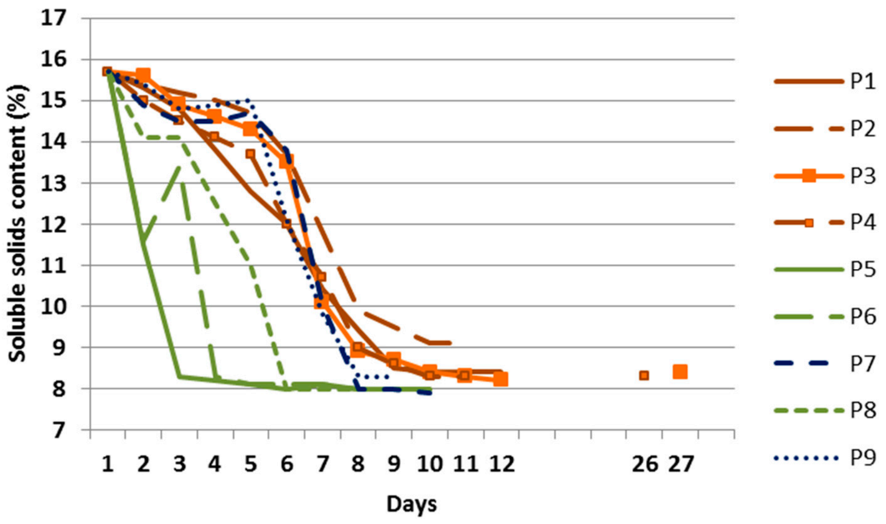

(b)

Figure S4. Kinetics of alcoholic fermentation of plum mashes from 'Čačanska Lepotica' variety. (a) Alcoholic fermentation of mashes from sound fruits (G1-G2) and heat-damaged fruits (P1-P2) processed by the same methods; (b) alcoholic fermentation of mashes from heat damaged. fruits (P1-P9) processed by nine various methods. All fermentations are performed in triplicate and mean values of soluble solids content were shown (standard deviations were omitted for clarity).

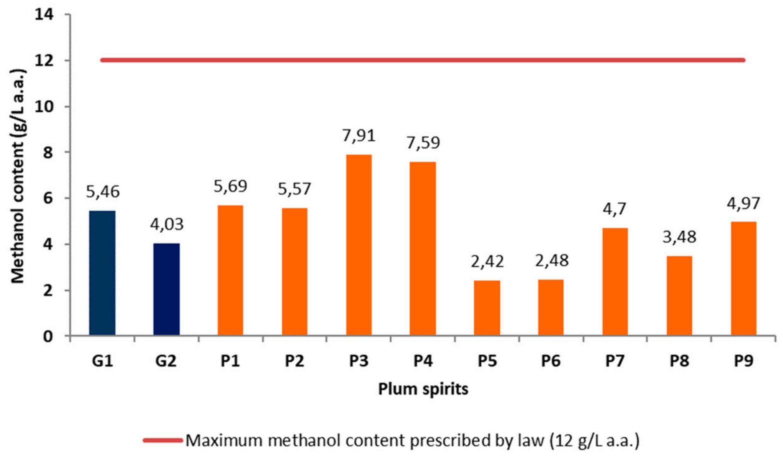

(a)

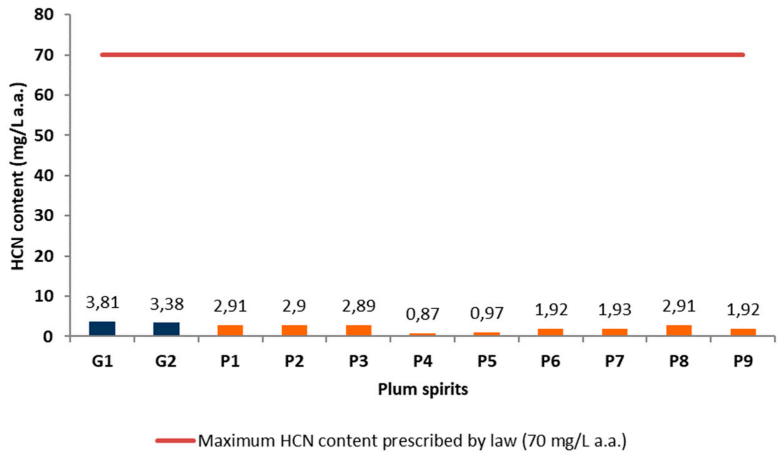

(b)

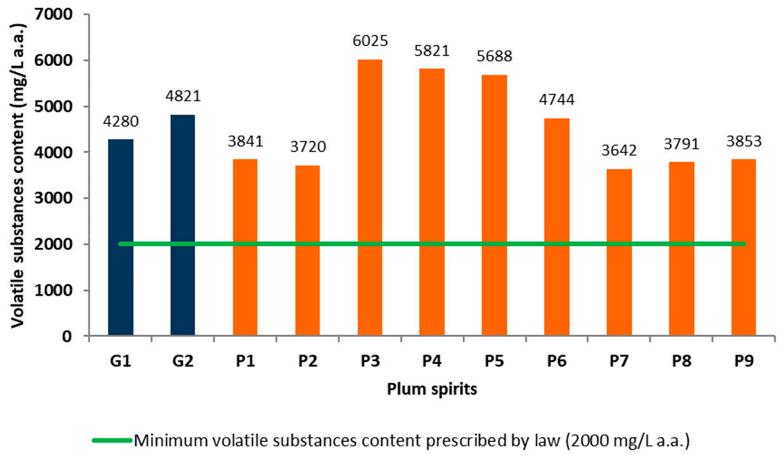

(c)

Figure S5. Content of components prescribed by EU regulation in plum spirits produced from sound plums, the Gleđica locality (G1-G2), and from heat-damaged plums, the Preljina locality (P1-P9). (a) methanol contents (g/L a.a.), (b) HCN contents (mg/L a.a.), (c) volatile substances contents (mg/L a.a.).

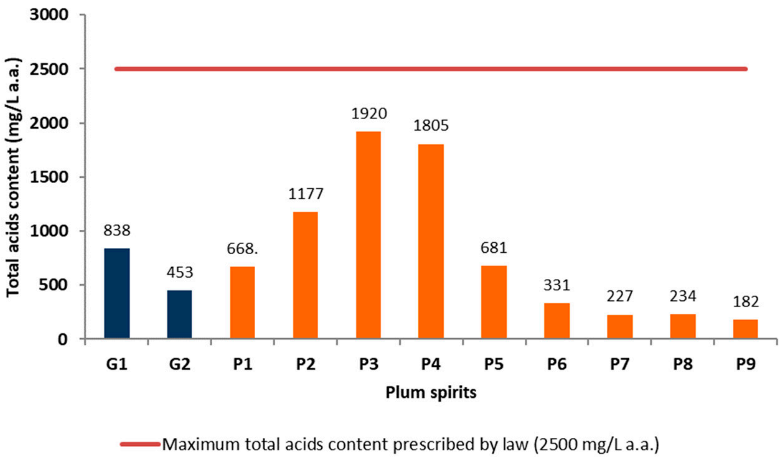

(a)

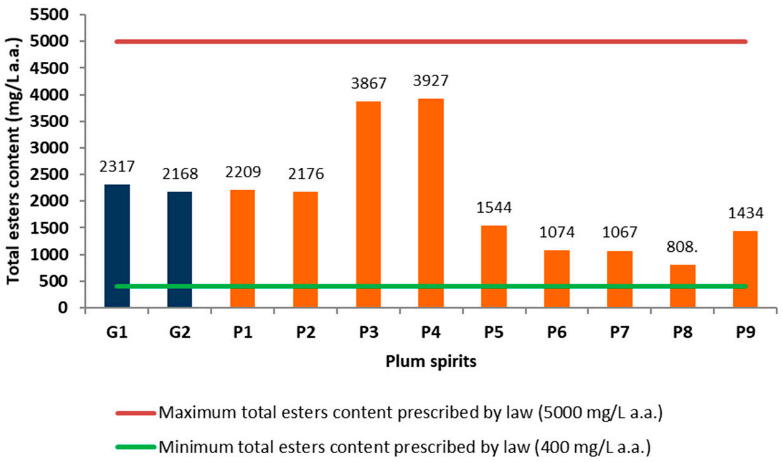

(b)

Figure S6. Content of components prescribed by old Serbian regulation in plum spirits produced from sound plums, the Gleđica locality (G1-G2), and from heat-damaged plums, the Preljina locality (P1-P9). (a) total acids content (g/L a.a.), (b) total esters content (mg/L a.a.).

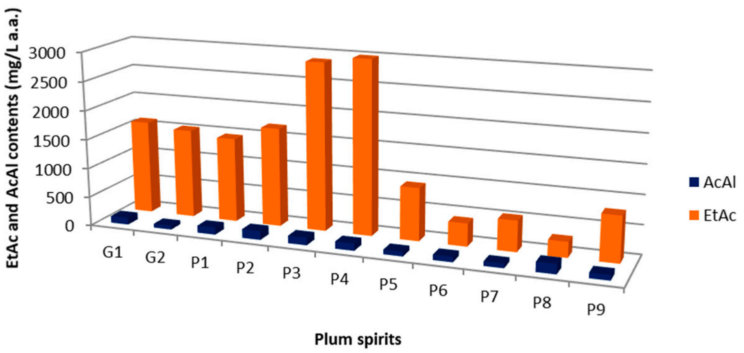

(a)

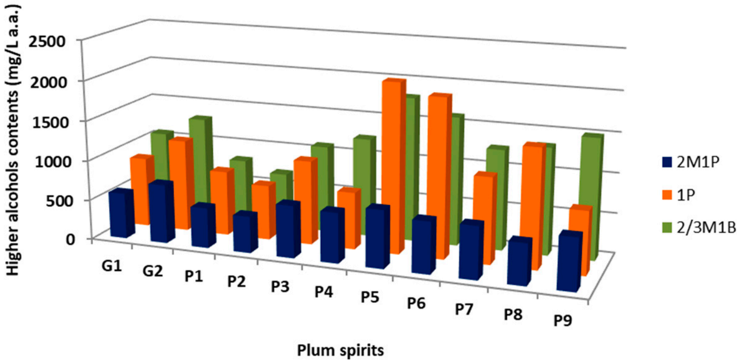

(b)

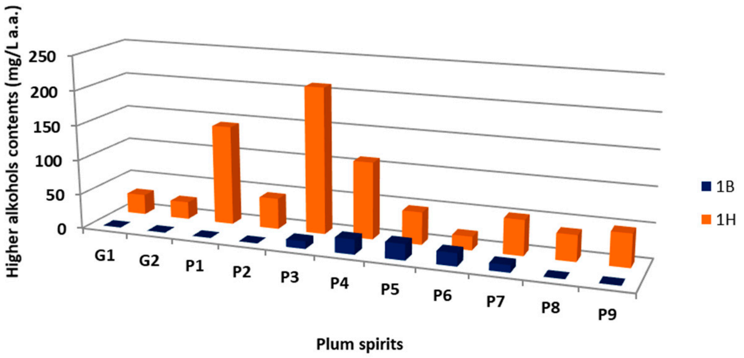

(c)

Figure S7. Profiles of major volatile components in plum spirits produced from sound plums, the Gleđica locality (G1-G2), and from heat-damaged plums, the Preljina locality (P1-P9) analyzed by GC-FID. (a) ethyl acetate (EtAc) and acetaldehyde (AcAl), (b) 2/3-methyl-1-butanol (2/3M1B), 1-propanol (1P) and 2-methyl-1-propanol (2M1P), (c) 1-hexanol (1H) and 1-butanol (1B).

Table S1. Odor activity values (OAVs) of volatile compounds in plum spirits obtained from sound fruits (G1-G2) and heat-damaged fruits (P1-P9) of ‘Čačanska Lepotica’

Compounds	OT (mg/L)	Odor descriptor[a, d, e, i, j]	OAV										
			G1	G2	P1	P2	P3	P4	P5	P6	P7	P8	P9
Alcohols													
1-Propanol	54[a]	Alcoholic, fermented, fusel, musty, yeasty, sweet, fruity, apple	0.66	0.63	0.33	0.29	0.49	0.35	1.08	0.99	0.55	0.83	0.39
2-Methyl-1-propanol	28.3[b]	Ethereal, winey, malty	2.89	2.93	1.57	1.51	2.20	2.21	2.60	2.28	2.53	2.05	2.32
1-Butanol	2.73[b]	Fusel, oily, sweet, balsamic, whiskey, malty, solvent	0.33	0.23	0.30	0.29	0.37	0.46	0.83	0.72	0.38	0.40	0.36
2/3-Methyl-1 butanol	179[b]	Fusel, alcoholic, winey, whisky, leathery, fatty, fruity, banana, pungent	1.82	1.92	1.00	0.90	1.37	1.64	2.38	2.12	1.88	1.96	1.99
1-Pentanol	1.5[c]	Fusel, oily, sweet, balsamic, pungent, fermented, bready, solvent	-	-	-	-	0.85	0.63	-	-	0.27	-	-
1-Hexanol	5.37[b]	Ethereal, fusel, oily, fruity, alcoholic, sweet, green, pungent	0.21	0.20	0.42	0.38	0.48	0.42	0.38	0.22	2.20	1.42	1.23
Z-3-Hexenol	1.257[d]	Green	-	-	0.47	0.33	0.46	0.35	0.40	-	2.08	1.45	0.88
1-Heptanol	26.6[a]	Musty, leafy, violet, herbal, green, sweet, woody, peony	-	0.01	0.01	0.01	-	0.02	0.02	0.02	0.02	0.01	0.01
1-Octanol	1.1[a]	Waxy, green, orange, aldehydic, roses, mushroom, floral, fatty	0.50	0.71	0.63	0.73	0.64	0.85	1.14	0.94	0.60	0.66	0.52
1-Nonanol	1.0[c]	Fresh, clean, fatty, floral, roses, orange, dusty, wet, oily	2.45	2.29	2.90	3.71	4.70	5.71	2.92	1.73	3.24	2.60	2.31
2-Phenylethanol	28.9[b]	Floral, roses, dried roses, sweet, fresh, bready, honey	0.20	0.26	0.15	0.14	0.17	0.15	0.17	0.17	0.19	0.16	0.20
Benzyl alcohol	40.9[a]	Sweet, floral scent, slightly balsamic, faintly almond-like, fruity	0.04	0.01	0.03	0.03	0.10	0.04	0.07	0.04	0.01	0.01	-
Esters													
Ethyl acetate	32.6[b]	Ethereal, fruity, sweet, weedy, green, pineapple,	11.41	9.94	7.53	9.34	14.35	16.33	5.48	2.14	3.16	1.20	4.27

		solvent											
Ethyl propanoate	19[b]	Sweet, fruity, rummy, pineapple, ethereal, winey, rum	0.03	0.03	0.03	0.02	0.06	0.04	0.03	0.02	0.02	0.01	0.02
Ethyl hexanoate	0.03[e]	Sweet, fruity, pineapple, waxy, green, banana	20.67	19.00	18.67	10.33	30.67	34.33	73.00	62.33	37.00	47.33	44.33
Ethyl octanoate	0.0129[b]	Fruity, winey, waxy, sweet, apricot, brandy, pear, creamy,cognac	194.57	204.65	153.49	137.98	272.09	287.60	689.15	514.73	327.13	513.18	351.94
Ethyl decanoate	1.12[b]	Sweet, waxy, fruity, apple, grape, oily, brandy, fatty, nut	2.75	3.15	2.06	2.37	3.31	3.38	8.05	5.10	4.80	6.23	4.91
Ethyl lactate	128[b]	Sharp, tart, fruity, buttery, butterscotch, sweet, acidic, Ethereal	0.04	0.03	0.15	0.04	0.22	0.12	0.04	0.02	0.03	0.02	0.03
Ethyl benzoate	1.43[b]	Fruity, medicinal, cherry	1.43	0.52	1.45	1.89	2.75	2.31	1.43	1.24	1.29	1.13	0.83
Ethyl cinnamate	0.0008[e]	Fruity, sweet, cinnamon-like, fruity-balsamic	4250.00	3200.00	512.50	712.50	-	375.00	787.50	887.50	287.50	462.50	-
Methyl acetate	>500[f]	Fruity, fragrant, sweet, solvent-like, estry, fruity, winey, cognac	0.01	0.00	0.00	0.00	0.01	0.01	0.00	0.00	0.00	0.00	0.00
Propyl acetate	4.7[g]	Solvent, celery, fruity, fusel, raspberry, pear, pungent, sweet	0.16	0.21	0.10	0.11	0.25	0.19	0.17	0.14	0.07	0.07	0.08
Isoamyl acetate	0.245[e]	Sweet, fruity, banana, solvent, ripe	6.94	9.39	8.00	3.51	8.12	9.35	14.65	26.24	21.27	31.10	26.29
Phenylethyl acetate	0.108[e]	Floral, roses, sweet, honey, fruity, tropical, yeasty, balsamic	-	4.44	4.07	3.24	3.06	4.26	4.81	8.15	26.94	9.81	16.48
Aldehydes													
Acetaldehyde	19.2[e]	Pungent, ethereal, aldehydic, fruity, musty, green apple	0.05	0.03	0.05	0.05	0.03	0.04	0.03	0.02	0.04	0.05	0.09
Hexanal	0.158[e]	Green, grassy	-	-	1.71	1.90	2.03	2.15	-	-	-	2.22	1.71
Nonanal	0.122[b]	Waxy, roses, fresh, orange/lemon peel, citrus, green, cucumber	13.28	13.36	11.15	13.69	14.67	13.11	13.11	10.74	8.03	14.02	13.52
Furfural	44[b]	Sweet, woody, almond, bread, baked, caramellike, phenolic	0.15	0.12	0.05	0.09	0.10	0.08	0.03	0.03	0.06	0.04	0.07
Benzaldehyde	4.2[b]	Sharp, sweet, bitter, almond, cherry, fruity, nutty	3.12	0.07	0.80	0.11	5.61	0.15	0.89	0.10	0.06	0.09	-
Heptanal	0.41[a]	Fresh, aldehydic, fatty, green, herbal, cognac, ozone	-	-	0.49	0.66	0.61	0.63	-	-	-	-	-
Acetals													

1,1-Ethoxy, methoxyetane	/	Sweet, fruity, or ether-like, pear drops, apple	-	-	-	-	-	-	-	-	-	-	-
1,1-Diethoxyetane	0.719[c]	Fruity, green, nutty, earthy, sweet, vegetable, ethereal	25.55	14.42	22.11	20.90	19.96	21.68	15.37	16.29	13.82	29.43	16.02
1,1-Diethoxy-hexane	/	Cognac, pear, floral, hyacinth, apple, fruity	-	-	-	-	-	-	-	-	-	-	-
1,1-Diethoxy-nonane	/	Aldehydic, floral, rosy, fruity notes, waxy	-	-	-	-	-	-	-	-	-	-	-
Acids													
Acetic acid	160[b]	Sharp, pungent, sour, vinegar	0.02	0.01	0.02	0.03	0.04	0.04	0.01	0.00	0.01	0.00	0.01
Hexanoic acid	2.52[b]	Sour, fatty, sweaty, cheesy	0.63	0.83	0.55	0.32	0.71	0.80	2.68	2.30	1.41	2.04	1.50
Octanoic acid	2.7[a]	Fatty, waxy, rancid, oily, vegetable, cheesy	2.42	3.33	2.58	1.29	2.81	3.61	11.78	10.62	8.16	12.03	8.62
Decanoic acid	2.8[e]	Rancid, sour, fatty, citrus	3.33	4.56	3.66	2.08	3.35	4.14	11.37	10.15	10.34	11.94	9.83
Dodecanoic acid	1.0[h]	Mild, fatty, soapy scent, reminiscent of bay oil or coconut	2.51	3.65	3.52	2.62	2.56	2.65	4.28	3.80	5.99	4.47	5.49

[a] [1]; [b] [2]; [c] [3]; [d] [4]; [e] [5]; [f] [6]; [g] [7]; [h] [8]; [i] [9]; [j] [10]; OT–Odour threshold; /–Unknown; -: means that OAV is not calculated.

Table S2. Sensory ratings of plum spirits produced from sound (G1-G2) and heat-damaged fruits (P1-P2)

Characteristics	G1	G2	P1	P2	P3	P4	P5	P6	P7	P8	P9
Colour	2.00±0.00	2.00±0.00	2.00±0.00	2.00±0.00	2.00±0.00	2.00±0.00	2.00±0.00	2.00±0.00	2.00±0.00	2.00±0.00	2.00±0.00
(max. 2 points)	aA	aA	aA	aA	A	A	A	A	A	A	A
Clearness	1.00±0.00	1.00±0.00	1.00±0.00	1.00±0.00	1.00±0.00	1.00±0.00	1.00±0.00	1.00±0.00	1.00±0.00	1.00±0.00	1.00±0.00
(max. 1 points)	aA	aA	aA	aA	A	A	A	A	A	A	A
Odor	6.13±0.22	6.18±0.19	6.11±0.18	6.03±0.17	5.63±0.39	5.68±0.33	5.85±0.46	6.18±0.26	6.10±0.27	5.86±0.52	6.21±0.23
(max. 7 points)	aA	aA	aA	aA	A	A	A	A	A	A	A
Taste	8.34±0.34	8.35±0.39	8.23±0.45	7.96±0.26	8.28±0.39	8.28±0.26	8.31±0.34	8.45±0.30	8.41±0.28	8.43±0.19	8.49±0.21
(max. 10 points)	aA	aA	aA	aA	A	A	A	A	A	A	A
Total	17.46±0.17	17.53±0.22	17.34±0.34	16.99±0.28	16.90±0.22	16.95±0.10	17.17±0.35	17.63±0.26	17.51±0.17	17.29±0.54	17.70±0.08
(max. 20 points)	aABC	aABC	aABC	aBC	C	BC	ABC	AB	ABC	ABC	A

Note: Different lowercase letters in the same row indicate statistically significant differences among the four samples (G1, G2, P1, and P2) produced from sound and heat-damaged fruits of 'Čačanska Lepotica' (Tukey's test, $p < 0.05$); Different uppercase letters in the

same row indicate statistically significant differences among all plum spirit samples (G1-G2 and P1-P9) (Tukey's test, $p < 0.05$). Values of all sensory characteristics are shown as mean ± standard deviation.

References

1. Fan, H.; Fan, W; Xu, Y. Characterization of key odorants in Chinese Chixiang aroma-type liquor by gas chromatography–olfactometry, quantitative measurements, aroma recombination, and omission studies. *J. Agric. Food Chem.***2015**, *63*, 3660–3668. https://doi.org/10.1021/jf506238f
2. Gao, W.; Fan, W.; Xu, Y. Characterization of the key odorants in light aroma type Chinese liquor by gas chromatography–olfactometry, quantitative measurements, aroma recombination, and omission studies. *J. Agric. Food Chem.***2014**, *62*, 5796–5804. https://doi.org/10.1021/jf501214c
3. Zheng, J.; Liang, R.; Huang, J.; Zhou, R.; Chen, Z.; Wu, C.; Zhou, R.; Liao, X. Volatile compounds of raw spirits from different distilling stages of Luzhou-flavor spirit. *Food Sci. Technol. Res.***2014**, *20*, 283-293. https://doi.org/10.3136/fstr.20.283
4. Uselmann, V.; Schieberle, P. Decoding the combinatorial aroma code of a commercial Cognac by application of the sensomics concept and first insights into differences from a German brandy. *J. Agric. Food Chem.***2015**, *63*, 1948–1956. https://doi.org/10.1021/jf506307x
5. Willner, B.; Granvogl, M.; Schieberle, P. Characterization of the key aroma compounds in Bartlett pear brandies by means of the sensomics concept. *J. Agric. Food Chem.***2013**, *61*, 9583–9593. https://doi.org/10.1021/jf403024t
6. Cacho, J.; Moncayo, L.; Palma, J.C.; Ferreira, V.; Cullere, L. The impact of grape variety on the aromatic chemical composition of non-aromatic Peruvian pisco. *Food Res. Int.***2013**, *54* 373–381. http://dx.doi.org/10.1016/j.foodres.2013.07.019
7. Xue, F.H.; Zhou, J.Q.; Yang, L.X. A comprehensive review of quantified flavour components in Chinese baijiu. *Int. Food Res. J.***2024**, *31*, 276–305. https://doi.org/10.47836/ifrj.31.2.02
8. Bordeu, E.; Agosin, E.; Casaubon, G. Pisco: production, flavor chemistry, sensory analysis and product development. In *Alcoholic beverages: Sensory evaluation and consumer research*. Piggott, J., Ed.; Woodhead Publishing Limited, Cambridge, UK, 2012; pp. 331–347. https://doi.org/10.1016/B978-0-85709-051-5.50016-X
9. Ratkovich, N.; Esser, C.; de Resende Machado, A.M.; Mendes, B.d.A.; Cardoso, M.d.G. The Spirit of Cachaça Production: An Umbrella Review of Processes, Flavour, Contaminants and Quality Improvement. *Foods***2023**, *12*, 3325. https://doi.org/10.3390/foods12173325
10. Christoph, N.; Bauer-Christoph, C. Flavour of spirit drinks: raw materials, fermentation, distillation, and aging. In *Flavour and fragrances*. Berger, R.D., Ed.; Springer-Verlag, Berlin, Heidelberg, Germany, 2007; pp. 219–239. https://doi.org/10.1007/978-3-540-49339-6_10
